# Google Goes Cancer: Improving Outcome Prediction for Cancer Patients by Network-Based Ranking of Marker Genes

**DOI:** 10.1371/journal.pcbi.1002511

**Published:** 2012-05-17

**Authors:** Christof Winter, Glen Kristiansen, Stephan Kersting, Janine Roy, Daniela Aust, Thomas Knösel, Petra Rümmele, Beatrix Jahnke, Vera Hentrich, Felix Rückert, Marco Niedergethmann, Wilko Weichert, Marcus Bahra, Hans J. Schlitt, Utz Settmacher, Helmut Friess, Markus Büchler, Hans-Detlev Saeger, Michael Schroeder, Christian Pilarsky, Robert Grützmann

**Affiliations:** 1 Department of Bioinformatics, Biotechnology Center, Technische Universität Dresden, Dresden, Germany; 2 Institute of Pathology, Universitätsspital Zürich, Zurich, Switzerland; 3 Department of Visceral, Thoracic, and Vascular Surgery, University Hospital Carl Gustav Carus, Dresden, Germany; 4 Institute of Pathology, University Hospital Carl Gustav Carus, Dresden, Germany; 5 Institute of Pathology, University of Jena, Jena, Germany; 6 Institute of Pathology, University of Regensburg, Regensburg, Germany; 7 Department of Surgery, University Hospital Mannheim, Mannheim, Germany; 8 Department of Pathology, Charité, Berlin, Germany; 9 Department of Surgery, Charité, Berlin, Germany; 10 Department of Surgery, University Hospital Regensburg, Regensburg, Germany; 11 Department of Surgery, University Hospital Jena, Jena, Germany; 12 Department of Surgery, Technische Universität München, München, Germany; 13 Department of Surgery, University of Heidelberg, Heidelberg, Germany; Tufts University, United States of America

## Abstract

Predicting the clinical outcome of cancer patients based on the expression of marker genes in their tumors has received increasing interest in the past decade. Accurate predictors of outcome and response to therapy could be used to personalize and thereby improve therapy. However, state of the art methods used so far often found marker genes with limited prediction accuracy, limited reproducibility, and unclear biological relevance. To address this problem, we developed a novel computational approach to identify genes prognostic for outcome that couples gene expression measurements from primary tumor samples with a network of known relationships between the genes. Our approach ranks genes according to their prognostic relevance using both expression and network information in a manner similar to Google's PageRank. We applied this method to gene expression profiles which we obtained from 30 patients with pancreatic cancer, and identified seven candidate marker genes prognostic for outcome. Compared to genes found with state of the art methods, such as Pearson correlation of gene expression with survival time, we improve the prediction accuracy by up to 7%. Accuracies were assessed using support vector machine classifiers and Monte Carlo cross-validation. We then validated the prognostic value of our seven candidate markers using immunohistochemistry on an independent set of 412 pancreatic cancer samples. Notably, signatures derived from our candidate markers were independently predictive of outcome and superior to established clinical prognostic factors such as grade, tumor size, and nodal status. As the amount of genomic data of individual tumors grows rapidly, our algorithm meets the need for powerful computational approaches that are key to exploit these data for personalized cancer therapies in clinical practice.

## Author Summary

Why do some people with the same type of cancer die early and some live long? Apart from influences from the environment and personal lifestyle, we believe that differences in the individual tumor genome account for different survival times. Recently, powerful methods have become available to systematically read genomic information of patient samples. The major remaining challenge is how to spot, among the thousands of changes, those few that are relevant for tumor aggressiveness and thereby affecting patient survival. Here, we make use of the fact that genes and proteins in a cell never act alone, but form a network of interactions. Finding the relevant information in big networks of web documents and hyperlinks has been mastered by Google with their PageRank algorithm. Similar to PageRank, we have developed an algorithm that can identify genes that are better indicators for survival than genes found by traditional algorithms. Our method can aid the clinician in deciding if a patient should receive chemotherapy or not. Reliable prediction of survival and response to therapy based on molecular markers bears a great potential to improve and personalize patient therapies in the future.

## Introduction

In the past decade, several studies have used microarray gene expression data from tumors to predict the clinical outcome of patients (see **Table S1**). The tumors included breast cancer [1]–[5], lung cancer [6]–[8], lymphomas [9]–[13], leukemia [14], [15], and others [16]–[18]. Outcome is usually measured by categorical, often binary variables such as survival up to a certain time, recurrence of tumor or metastasis before a certain time, or success of treatment. Predicting such variables from gene expression levels can be viewed as a classification problem, and the set of genes used for prediction is commonly referred to as a signature. Accurate outcome prediction can be used clinically to select the best of several available therapies for a cancer patient. For instance, a low risk patient can be advised to select a less radical therapy.

Whereas differences in gene expression between tumor and healthy tissue or between different tumor tissues are often strong, gene expression differences between patients with the same type of tumor but different outcome are more subtle. For example, distinguishing acute myeloid from acute lymphoblastic leukemia has been demonstrated to be up to 100% accurate using only a few genes [19]–[21]. In contrast, outcome prediction is a much harder problem, with classification accuracies commonly in the range of 50–70%. It is therefore not surprising that many studies suffer from one or several of the following three problems: (i) limited or overoptimistic prediction accuracy, (ii) limited reproducibility, and (iii) unclear biological relevance of the genes used for prediction. For example, an early study predicting breast cancer metastasis using 70 genes [3] was subsequently found (i) to have lower than initially published predictive accuracy on the same or independent data sets [22], [23], (ii) to be difficult to reproduce [24], and (iii) to have used 70 genes that can be easily replaced by 70 different but equally predictive genes derived from the same data, questioning the biological relevance of the particular 70 genes of the original study [25]. Notably, predictive gene sets derived from different studies for the same disease show almost zero overlap, questioning their biological relevance.

To address and overcome these problems we have developed a computational network-based strategy for outcome prediction. Our algorithm, NetRank, couples gene expression measurements with a network of known relationships between the genes' products. NetRank is based on Google's PageRank algorithm [26]. PageRank uses the hyperlink information between web documents to better decide which documents are the most relevant ones. Similarly, NetRank uses biological interaction information between genes' products to better decide which genes are the most relevant for outcome prediction. Such interaction information is available in protein–protein, transcription factor–target, or gene co-expression networks. The inclusion of network information serves two purposes. First, gene products with many interactions should have a higher biological relevance since they can exert a bigger influence on a biological system. Second, considering network neighbors can help the algorithm to ignore correlations between expression and outcome that have no underlying biological causality. Such correlations can arise simply by chance, often due to the fact that microarray measurements are noisy and that the number of samples is typically several orders of magnitudes smaller than the number of genes investigated.

We wanted to test the NetRank idea on outcome prediction for pancreatic cancer, for which no microarray-derived signature was yet published. Pancreatic ductal adenocarcinoma accounts for approximately 130,000 deaths each year in Europe and the United States [27], [28]. It has an extremely poor prognosis with a 5-year survival rate below 2% [29], [30]. Currently, only a few prognostic factors for pancreatic cancer survival are used in the clinical setting, among them CA 19-9, alkaline phosphatase, LDH, levels of white blood cells, aspartate transaminase, and blood urea nitrogen [31]. A considerable number of protein markers for pancreatic cancer prognosis have been investigated using immunohistochemistry [32]. However, the clinical value of most of these markers remains to be determined, and also most of these markers were found by chance or educated guesses rather than a systematic, genome-wide approach.

The aim of our study was therefore (i) to carry out a genome-wide screen for genes whose expression in pancreatic cancer tissue samples reliably correlates with the patient survival time, and (ii) to use these genes as a molecular signature for reliable survival prediction. To this end, we collected and analyzed tissue samples from patients with pancreatic ductal adenocarcinoma from Germany in a multi-center study. Applying NetRank to gene expression profiles of these samples identified seven candidate marker genes prognostic for outcome. To assess the clinical value of our identified marker genes, we validated them on an independent patient cohort. We found that signatures based on these markers were more accurate than traditional clinical parameters and more accurate than signatures identified with other computational approaches.

## Results

We obtained gene expression profiles of sufficient quality from 30 pancreatic ductal adenocarcinoma samples of patients that underwent surgery in German university hospitals between 1996 and 2007, hereafter referred to as the screening dataset (see Materials and Methods for quality criteria and experimental details). For each patient, the clinical parameters age, sex, cancer staging according to the tumor-node-metastasis (TNM) classification, and survival time after operation were recorded. **Table 1** shows an overview of the patient characteristics. For predicting the prognosis of a patient, we assigned patient samples to either a poor or a good prognosis group depending on patient survival time. Such an assignment is straightforward if the survival time is bimodally distributed. However, such a bimodal distribution is often absent in cancer patient survival times (see **Figure S1**). In this case, a common choice is to split by the median survival time independently of the distribution (see for example [13], [33]), which has the advantage of yielding two classes of equal size. Thus, we defined prognosis as poor if the patient survival time was less than the median survival time of 17.5 months, and as good otherwise. This resulted in two prognosis groups with 15 patient samples each. The goal was to identify a signature of genes whose expression levels allowed to correctly predict the prognosis group of a patient. Since we wanted the resulting signature to be applicable in a clinical setting using immunohistochemistry staining of the signature proteins, we opted for a signature size in the order of five to ten genes.

**Table 1:** Clinical characteristics of patients used in this study.

	Screening dataset		Validation dataset		Validation dataset	
			Adjuvant therapy		No adjuvant therapy	
	n	%	n	%	n	%
Total number of patients	30	100	172	100	240	100
Sex						
female	19	63	73	42	115	48
male	11	37	99	58	125	52
Age						
Median (years)	65		65		66	
Range (years)	40–82		33–81		31–85	
Patient survival						
1 year	17	57	104	60	146	61
2 years	9	30	38	22	83	35
3 years	6	20	12	7	54	23
4 years	3	10	4	2	32	13
5 years	0	0	3	2	15	6
Median (months)	17.5		14.4		15.5	
Range (months)	5–53		1–109		1–189	
Primary tumor†						
pT1	0	0	3	2	6	2
pT2	2	7	28	16	42	18
pT3	26	87	137	80	182	76
pT4	2	7	4	2	9	4
Regional lymph nodes†						
pN0	12	40	43	25	88	37
pN1	18	60	129	75	148	62
Distant metastasis						
M0	29	97	152	88	182	76
M1	1	3	11	6	17	7
Histologic grade						
G1	1	3	7	4	17	7
G2	11	37	67	39	108	45
G3	18	60	97	56	111	46
Residual tumor						
R0	24	80	119	69	167	70
R1	3	10	40	23	57	24
R2	3	10	6	3	5	2
Stage grouping‡						
I	2	7	11	6	18	8
IIA	10	33	26	15	54	22
IIB	15	50	112	65	106	44
III	2	7	3	2	4	2
IV	1	3	11	6	17	7

The screening dataset (genome-wide gene expression profiling) comprises 30 samples of surgically resected pancreatic ductal adenocarcinoma from patients without adjuvant chemotherapy. The validation dataset (immunohistochemistry of seven marker candidates) comprises samples from 412 patients, of which 172 had received adjuvant therapy and 240 had not. Significant differences between the adjuvant and no adjuvant therapy subgroups were found for regional lymph nodes status (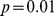, Fisher's exact test) and for the stage groupings (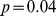, Fisher's exact test). Differences in all other variables were not significant.

**†:** Based on postsurgical histopathological assessment (indicated by the p prefix).

**‡:** Stage was assessed by the American Joint Committee on Cancer 2006 guidelines.

### Signature evaluation methodology

**Figure 1:**
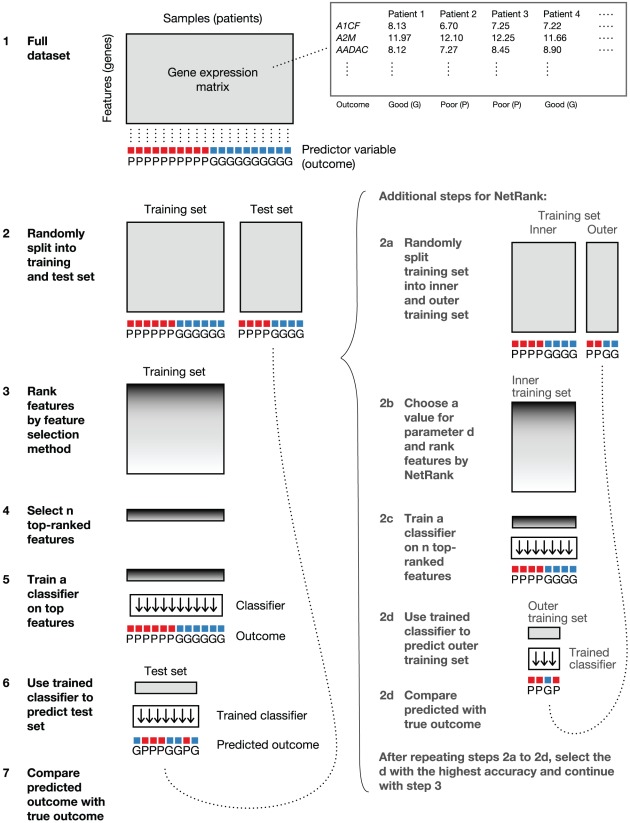
Monte Carlo cross-validation workflow to evaluate the accuracy of methods for ranking genes for outcome prediction. The full dataset is a gene expression matrix with 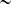
8,000 features (the genes) as rows and 30 samples (the patients) as columns. For each patient, the outcome (poor or good) is given (1). The dataset is randomly divided into a training and a test set (2). Within the training set, genes are ranked by how different they are between patients with poor and good outcome (3). The most different genes are selected (4). They are used to train a machine learning classifier on the training set (5). After training, the classifier is asked to predict the outcome of the test set patients (6). The predicted outcome is compared with the true outcome and the number of correctly classified patients is noted (7). Steps 2–7 are repeated 1,000 times, and the resulting final accuracy is obtained by averaging over the 1,000 accuracies of step 7.

To identify signature genes, various state of the art methods exist. To evaluate these methods in comparison to our own NetRank method on the screening dataset, the following workflow was employed (see **Figure 1**). After filtering out low expression and low variance genes, 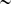
8,000 genes remained as potential signature genes. Five different methods for ranking genes according to their power to discriminate between the two prognosis groups were tested: (i) fold change, as defined by the ratio of a gene's mean expression in one group over the other group, (ii) the t-statistic, (iii) Pearson and Spearman rank correlation coefficients of a gene's expression with the survival time of the patient, (iv) the SAM (Significance Analysis of Microarrays) method [34], and (v) our NetRank algorithm (see Materials and Methods for details). In addition, selecting genes randomly was included as a control method. For each method, a support vector machine classifier was trained using the 5–10 top ranked genes as features. Prediction accuracy as defined as the percentage of correctly classified samples was evaluated with different training and test set sizes. All feature selection and machine learning steps were subjected to Monte Carlo cross-validation, which is a recommended and relatively un-biased evaluation strategy [22], [35] (see **Figure 1** and Materials and Methods for details).

### NetRank, a network-based approach for identifying predictive marker genes from microarray data

We introduce NetRank, a modified version of the PageRank algorithm [36]. As employed by the Google Internet search engine, the PageRank algorithm uses network information (hyperlinks) between documents in the world wide web to assess the relevance of a document. A document is important if it is highly cited by other documents. Moreover, citations from important documents have more weight than citations from unimportant documents. Thus, in order to measure the relative importance of a document within the set of all web documents, PageRank ranks a document according to the number of highly ranked documents that point to it. Similarly, NetRank assigns a score to a gene which is influenced by the scores of genes linked to it. This linkage can be defined in several ways. Morrison et al. [37] described an adaptation of PageRank which uses networks where genes are connected if they share a Gene Ontology annotation. Here, we employ known transcription factor–target relationships (from TRANSFAC [38]), protein–protein interaction (from HPRD [39]), and gene co-expression (from COXPRESdb [40]) to define three different gene–gene networks, which were used with NetRank. NetRank first assigns as a score for each gene the absolute correlation of its mRNA expression level with the patient survival time in the dataset. The network is then used to spread this correlation to its neighbors and beyond. The genes with the highest NetRank score are then selected as signature genes (see Materials and Methods for details).

### NetRank outperforms traditional approaches

We first compared the three above mentioned networks for NetRank and found that signatures obtained using the TRANSFAC transcription factor network consistently had higher predictive accuracies than those using the protein interaction or the co-expression network. We therefore decided to only use the TRANSFAC network for NetRank in the following experiments.

**Figure 2:**
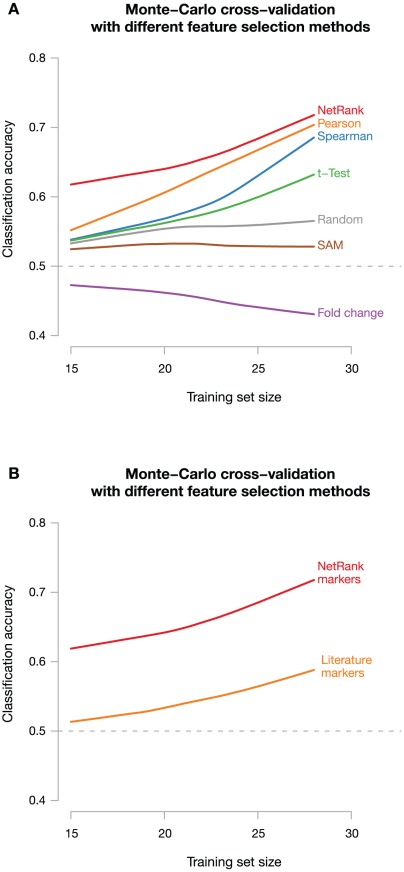
NetRank feature selection outperforms standard feature selection methods. (**A**) The accuracy of different feature selection methods for predicting patient outcome was tested on the screening dataset. The NetRank feature selection using a transcription factor network is shown in red. For smaller training set sizes, our method is superior to all other feature selection methods, reaching an accuracy of 72% in a Monte Carlo cross-validation. (**B**) Markers found with NetRank are more accurate than markers described in literature.

For all training set sizes, signatures selected with NetRank using the TRANSFAC transcription factor–target network showed higher predictive accuracies than those selected by any of the other four methods (**Figure 2A**). NetRank showed a maximum accuracy of 72% (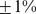 standard error of the mean, s.e.m.) with a training set of 28 samples and a signature size of 7 genes. This compares favorably to studies in other cancers, which show accuracies in the range of 50–70%. We found that NetRank is especially beneficial for small training set sizes, where there is a 7% (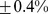 s.e.m.) improvement in accuracy compared to the Pearson correlation method.

Since many single prognostic markers for pancreatic cancer have been described in the literature, we next asked whether markers found with NetRank were superior to these literature markers. To this end, 51 markers identified via a literature search were used to train a support vector machine with different training set sizes (see Materials and Methods and **Table S2**). Surprisingly, we found that the NetRank markers showed on average a 12% higher accuracy than the literature markers (**Figure 2B**).

Using NetRank, we identified seven genes (*STAT3*, *FOS*, *JUN*, *SP1*, *CDX2*, *CEBPA*, and *BRCA1*) as most relevant for predicting survival in patients with pancreatic ductal adenocarcinoma. These seven marker candidates were validated in two ways: first, by quantitative RT-PCR of the screening dataset to confirm the microarray gene expression measurements, and second, by immunohistochemical analysis of protein levels in an independent dataset of 412 patients (the validation dataset, see **Table 1**).

### The marker genes control the expression of many target genes that are involved in cancer-related pathways

Of our seven markers, we found high expression to be associated with shorter survival for *STAT3*, *FOS*, and *JUN*, and high expression associated with longer survival for *SP1*, *CDX2*, *CEBPA*, and *BRCA1*. This is in line with most previous studies. *STAT3* is a well-known oncogene and persistently activated in many human cancers, including all major carcinomas [41]. *FOS* and *JUN*, which constitute the AP-1 transcription factor, have been linked to both tumor progression and suppression [42]. *SP1* was reported to be associated with poor prognosis in gastric cancer and recently also in pancreatic ductal adenocarcinoma [43], [44]. *BRCA1* is a DNA damage repair protein where loss-of-function mutations typically lead to early onset of breast cancer and ovarian cancer [45].

**Figure 3:**
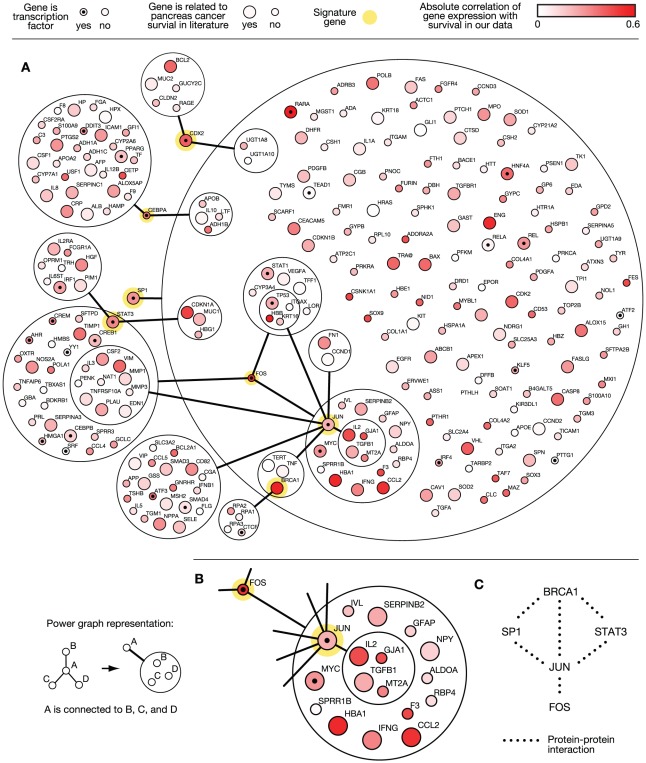
Regulatory network around signature genes. (**A**) All direct neighbors for the seven candidates STAT3, FOS, JUN, SP1, CDX2, CEBPA, and BRCA1 (marked yellow). Transcription factors are marked with a dot. Genes reported in the literature associated with pancreatic cancer survival according to GoGene are represented with larger circles. The absolute correlation coefficient of gene expression with survival in the screening dataset is shown in red. (**B**) Selection of the network showing genes that are regulated by FOS and SP1. It contains many literature-associated and highly correlated genes. (**C**) Protein–protein interactions among all signature genes, representing physical interactions between the transcription factors SP1, STAT3, JUN, FOS and the transcription coactivator BRCA1.

**Figure 3A** shows the direct network neighbors of the seven candidates. The network is shown in power graph representation, which reduces the number of edges drawn without information loss [46]. The underlying network of regulatory relationships was obtained from the TRANSFAC database [38]. The network shows that the seven markers (yellow) regulate a total of 323 targets. The correlation of the expression of a gene with the survival of the patient in the screening dataset is shown in red. Genes with larger circles were previously described in the literature as being associated with survival in pancreatic cancer.

The marker protein with the most regulatory interactions is the transcription factor SP1. Some of its targets are additionally regulated by other markers such as CEBPA, STAT3, FOS, and JUN. One interesting module defined by the genes that are regulated by SP1 and FOS is shown in **Figure 3B**. It contains many genes already known to be associated with survival in pancreatic cancer as well as some genes highly correlated with survival in our data, such as *HBA1*, *F3*, *CCL2*, *IL2*, and *GJA1*. A subset of this module is defined by genes that are also regulated by JUN. This subset contains the genes *IL2*, *TGFB1*, *MT2A*, and *GJA1*, which correlate well with survival.

Among the interaction partners of the markers, more than one-third has been previously reported as associated with survival or prognosis in pancreatic cancer, including PPARG, MUC4, and SMAD3 [47], [48]. A pathway analysis using KEGG [49] showed that 91 of the interacting genes are involved in signaling pathways, most prominently the MAPK and JAK-STAT signaling pathways. Furthermore, 53 of the interacting genes are involved in known KEGG cancer pathways (see **Table S3**).

### Two immunohistochemistry signatures predict survival for patients with and without adjuvant therapy

To validate our findings, we analyzed protein levels of our markers in an independent set of 412 patients (the validation dataset, see **Table 1**). We wanted to test how well the proteins encoded by the marker genes are indicative for the survival of a patient when assessed by immunohistochemical staining of the patient's tumor. Using tissue microarrays, immunohistochemistry stainings were obtained for each of the seven marker proteins STAT3, FOS, JUN, SP1, CDX2, CEBPA, and BRCA1 for each patient in the validation dataset (see **Figure S2**). The predictive accuracies of the marker staining intensities (encoded in two levels, low or high, see Materials and Methods) were evaluated after training a support vector machine classifier in a leave-one-out cross-validation procedure. The classifier predicted patients to belong either to a low risk (good prognosis) group, or to a high risk (poor prognosis) group. Using backward elimination, starting from the full set of markers, markers were removed one at a time until the accuracy of the trained classifier failed to improve. The clinical parameters tumor size (T), regional lymph nodes (N), distant metastasis (M), histological grade (G) and residual tumor (R) were tested in the same manner for comparison.

Since some patients in the validation dataset received adjuvant therapy (mostly chemotherapy with gemcitabine), and the adjuvant therapy had an influence on survival time (although not quite as expected, see **Figure S3**), we split the validation dataset into a group of patients with and without adjuvant therapy. Note that the decision to treat a patient with adjuvant chemotherapy is so far not based on any molecular markers (which was one motivation for our study). Chemotherapy is part of the standard treatment for pancreatic cancer in Germany since many years and is recommended for every patient. However, patients in a reduced state of health and patients who refuse it will not receive chemotherapy.

#### A signature for prognosis of patients with adjuvant therapy

**Figure 4:**
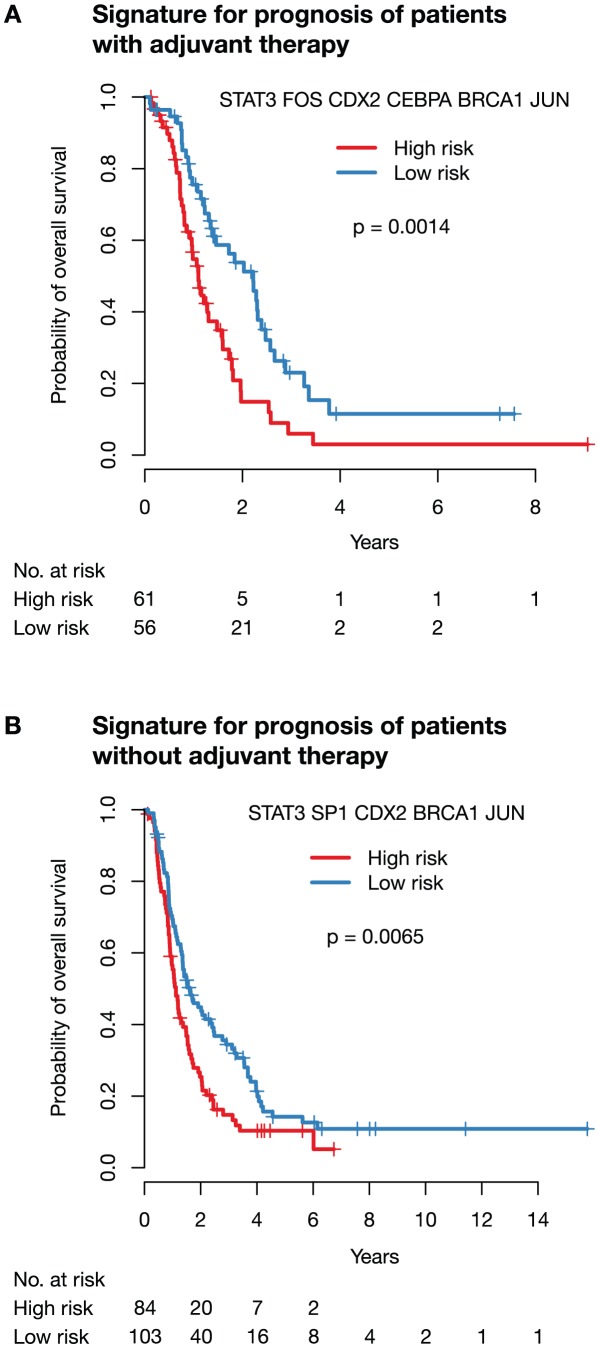
Signature to predict risk in patients with and without adjuvant therapy. (**A**) Signature to predict risk in patients with adjuvant therapy. The signature was developed with patients receiving adjuvant therapy separated by their median survival into two groups, a high risk group with shorter survival and a low risk group with longer survival. A classifier trained with the signature using leave-one-out cross-validation shows a significant difference between the predicted low and high risk group (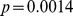, logrank test). (**B**) Signature to predict risk in patients without adjuvant therapy. The signature was developed with patients not receiving adjuvant therapy separated by their median survival into two groups, a high risk group with shorter survival and a low risk group with longer survival. A classifier trained with the signature using leave-one-out cross-validation shows a significant difference between the predicted low and high risk group (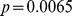, logrank test).

The signature with the highest predictive accuracy of 65% (68% area under the ROC curve, AUC) consisted of the proteins STAT3, FOS, JUN, CDX2, CEBPA, and BRCA1 (see **Figure 4A** and **Figure S4A**). The median survival time was 17 months in the predicted low-risk group and 12 months in the high-risk group (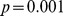, logrank test). The accuracy could be further improved by adding clinical parameters (T, N, M, G, R, see see **Table 1**) to the classifier. Starting with the combination of all protein and clinical markers and using again backward elimination, the combination of the markers STAT3, FOS, SP1, CEBPA, and BRCA1 with the clinical parameters R, N, and M achieved the highest prediction accuracy of 70% (69% AUC). The best combination of clinical parameters alone was G, R, and T with an accuracy of 61% (63% AUC).

#### A signature for prognosis of patients without adjuvant therapy

Using the same approach, we developed a signature for the subgroup of patients without adjuvant therapy. The marker signature with the highest predictive accuracy of 60% (59% AUC) consisted of STAT3, JUN, SP1, CDX2, and BRCA1 (see **Figure 4B** and **Figure S4B**). The median survival was 18 months in the predicted low-risk and 13 months in the predicted high-risk group (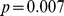, logrank test). The combination of the markers FOS and BRCA1 with the clinical parameters G, R, and M achieved a predictive accuracy of 65% (64% AUC). The best combination of clinical parameters alone was G, R, N, and M with an accuracy of 59% (64% AUC).

#### Signatures are superior to clinical parameters and independently predictive of outcome

Both signatures based on immunohistochemical staining of the tumor tissue improve prediction of patient prognosis. Comparison of the above signature accuracies shows that the additional predictive value of the signature markers compared to clinical parameters is 9% for patients with and 6% for patients without adjuvant therapy (70% versus 61% for adjuvant, and 65% versus 59% for non-adjuvant therapy).

To further ensure that the markers predict survival independently from clinical parameters, we tested if there was a significant interaction in a Cox proportional hazard model for any pair of a marker and a clinical parameter. We did not find a significant interaction between any marker and any clinical parameter (N, M, G, R) used in our signatures. Hence, the markers found in our study are independently predictive of outcome and superior to established clinical parameters.

One important finding of our validation was that the accuracy dropped from 72% to 65% when going from mRNA to protein level. Two reasons are likely to contribute to this fact. First, protein levels are known to not always correlate well with mRNA levels [50], [51]. Second, protein levels were encoded based on immunohistochemistry staining intensity in only two grades (low, high), leading to a reduction of information available for the classifier compared to the continuous microarray measurements.

### Comparison with other studies

The accuracies of our signatures are comparable to those found in other cancer studies. Stratford et al. [18] found six genes differentially expressed in tumors from pancreatic cancer patients with localized disease compared to metastatic disease using the significance analysis of microarrays (SAM) method [34]. Based on these six genes, they classified patients into high- and low-risk groups with 1-year survival rates of 55% and 91%, respectively. Our signatures classify patients into high- and low-risk groups with 1-year survival rates of 54% and 76%, respectively (adjuvant six-gene signature) and 55% and 69%, respectively (non-adjuvant five-gene signature). Unfortunately, Stratford et al. [18] did not report a classification accuracy percentage. Most surprisingly, although patients and methods were different, the six genes identified in their study and our seven genes share one gene of the Fos family. As mentioned before, there has hardly been any overlap among the signatures published so far for one tumor type. The discovery of *FOS* in both methods thus highlights its importance for tumor progression and outcome in pancreatic cancer, and further underlines the ability of our method to find reproducible and biologically significant markers.

### The influence of NetRank parameters on the results

NetRank depends on a number of parameters (see Materials and Methods for a full description): the choice of the genes' initial values 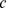 that spread through the network, the damping factor 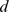 which influences the amount of spread, the choice of the network, and the role of noisy and uninformative genes, which are filtered out. Next, we investigate NetRank's dependence on these parameters.

#### Choice of genes' initial values

As mentioned above, NetRank spreads the correlation of a gene's mRNA level with survival time in our dataset through the network (see parameter 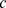 in Materials and Methods). What happens if the value 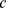 does not use this specific dataset-dependent fingerprint, but instead a constant value so that all genes are *a priori* equally important–or, from another perspective, if one only relies on network topology? We tested this and found a prediction accuracy of 62–65% (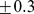
–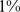 s.e.m., see **Table S4**) for all training set sizes, which is considerably less than the 72% (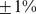 s.e.m.) maximum accuracy of the original NetRank. This is interesting since it implies that although some improvement is already gained by focusing on network hubs independent of gene correlations, there is indeed more prognostic information that comes from the use of concrete expression values linked to clinical data.

#### Network damping factor

Another important question regarding the algorithm relates to the optimal choice of the damping factor 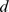 (see Materials and Methods) used to spread values through the network. Setting 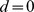 corresponds to no influence of the network and full influence of the gene expression data, whereas 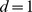 corresponds to full network influence and no influence of the gene expression data. According to [26], Google's PageRank uses 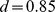.

To find the optimal 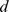 for our dataset, we added an inner cross-validation loop to our Monte Carlo workflow (see **Figure 1**, steps 2a to 2d). A value of 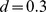 on the TRANSFAC network of transcription factors and their regulated targets gave the best results in terms of predictive accuracy of the identified signature genes. Is there an intuitive reason why 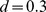 is the best choice? Unfortunately, there seems to be no optimal damping factor across different cancer microarray studies. The value 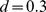 is optimal for this one study. We also have data for optimal d-values in ten other cancer studies [5], [13], [33], [52]–[58] and they vary (from 0.1 to 0.9, with 0.3 being the most frequent value). This suggests that in some cases more distant genes exert a stronger influence. Also, the incompleteness of current interaction networks implies that the fraction of disease-relevant genes covered by the network varies between diseases. For some studies, the network might cover the particular disease-relevant genes well and for others not. Thus, the d-value indicates also the match of the network's coverage and connectivity to the particular study.

#### Influence of direct neighbors

A damping factor 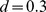 suggests that the results strongly depend on direct neighbors: The PageRank can be viewed as an indication of the likely location of a random surfer who iteratively traverses the network. At each iteration the surfer makes a step from one node to one of its neighbors with probability 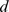, while with probability 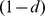 he makes a jump to a random node in the network. In NetRank such a random node is selected with probability proportional to the correlation of the corresponding gene expression with patient survival (given by vector 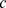). With 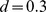, the probability of making two consecutive steps is 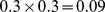. Thus, the final ranking is obtained with information that comes for more than 90% from initial correlation values and direct neighbors only.

So how does an algorithm perform that considers only direct neighbors instead of the whole network? In other words, is the global network structure needed to judge each gene, or is its local neighborhood sufficient? We implemented a variant of NetRank that spreads values only to direct neighbors. Each node is ranked according to the average of the initial node values of its direct neighbors. To our surprise, as shown in **Figure S5**, this direct neighbor variant performed almost identically to the ranking by Pearson correlation (without network information). Hence, at least for this study, it is important to also consider distant neighbors.

With a sufficiently large number of training samples, NetRank nearly always performs best, but the difference to Pearson correlation and the direct neighbor method becomes sufficiently small. With few training samples, NetRank and the network only (constant 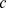 value) approach compete for the best accuracy. It seems that the strength of NetRank is to rely on network topology when data are sparse and correlation can be misleading, and to shift to relying on correlation when sufficient data is available.

#### Choice of network

The results presented here use a regulatory network, TRANSFAC. We also experimented with protein interaction data from the Human Protein Reference Database (HPRD) [39] and co-expressed genes from COXPRESdb [40]. Although the protein interaction and co-expression networks are much larger and could have better coverage, they led to worse results. This suggests that for outcome prediction direct regulation is more valuable information than general interaction or co-expression. In fact, for the ten other cancer outcome studies we investigated [5], [13], [33], [52]–[58], a TRANSFAC-based network gave the best results in seven, and a HPRD-based network in three of the studies.

#### Filtering

We initially filtered out low expression and low variance genes from our microarray data. It is commonly agreed upon that this is a necessary first step when searching for discriminative genes in microarray data. But since NetRank is a network-based, integrative approach, removing genes that could otherwise provide information for their neighboring nodes is probably a suboptimal strategy. For NetRank, we therefore keep all genes, but assign an initial value of zero to those genes that do not pass the filter. This leads for example to downranking of a node that has many neighbors with a value of zero, but it also allows for upranking of a node with an initial value of zero that has many high value neighbors.

The question if filtering is necessary at all however remains. One would expect NetRank to be robust against “noise” since it uses additional network information that can help to detect and ignore noise. To clarify, our initial filtering actually serves two purposes. It removes not only noise (genes with low expression that are most likely not expressed), but also removes uninformative genes (genes that have low variance and thus cannot be used to discriminate between any classes in the data). Note that uninformative genes can be highly expressed – in our data, one third of the genes have high expression, but low variance.

To separately assess the effect of noisy (low expression) and uninformative (low variance) genes on the accuracy we ran further experiments with subsets of our data. The results were quite interesting. When we did remove uninformative genes but not remove noisy genes, the resulting accuracy was still 71% (compared to 72% in the original filtering). This suggests that NetRank is rather robust to noise. However, when we did remove noisy genes but not remove uninformative genes, the accuracy dropped to 56%, which suggests that NetRank is not robust at all with respect to the presence of uninformative genes. Inspection of the highly ranked genes in this case revealed that the majority of them were uninformative high expression, low variance genes. As the top 5–10 genes are used for classification in our SVM, it is not surprising for the accuracy to drop if more than half of these genes are uninformative and hence cannot help in classification. A comparison with the original filtered approach showed that the previous top-ranked nodes are still found, but that the uninformative nodes score even higher.

So why does NetRank assign high scores to uninformative genes? There is a simple explanation: the input for NetRank are not use gene expression values, but the genes' correlation with survival. So besides the network, the basis for ranking is a gene's absolute correlation coefficient, not its expression value. When we plotted the distribution of these correlation coefficients in four different groups (low/high expression/variance), we found that the highly expressed uninformative genes had very similar correlation coefficients compared to informative genes (see **Figure S6**). This means that is virtually impossible for NetRank to detect if a gene will be uninformative for classification, as it can have an equally good correlation value than an informative gene. The fact that many highly expressed low variance genes are apparently correlated with survival suggests that either Pearson correlation is not an ideal measure here, or that the number of samples is too small to give a higher correlation signal in the informative genes. Since Spearman rank correlations show a similar pattern, we believe that the latter is true and that for this dataset size filtering is the best strategy.

To summarize, while NetRank is rather robust against noisy low expression measurements, it is essential to filter out uninformative genes (i.e. set their initial values to zero) before running NetRank.

## Discussion

Here, we present a novel method for identifying prognostic markers from genome-wide gene expression data. A key feature of the method is that it judges the relevance of a gene as marker not only by its expression (or rather the correlation of its expression with survival), but also by the expression of its neighbors. Thus, it can detect and therefore avoid markers that correlate with survival simply by chance or noisy measurements, but not due to an underlying biological causality. We applied this method to microarray data from 30 freshly frozen samples of pancreatic ductal adenocarcinoma and obtained a prognostic marker set of seven genes. This set showed an accuracy of 72% in predicting the prognosis of a patient. To ensure validity of this result, we employed a rigorous Monte Carlo cross-validation procedure. We then validated these genes using high-throughput immunohistochemistry of samples from surgically resected tumors from an independent cohort of 412 patients; roughly half of these received adjuvant therapy. From the marker set we derived a six-gene signature for patients with adjuvant therapy and a five-gene signature for patients without adjuvant therapy. Both signatures improve prediction of patient prognosis compared to the use of clinical parameters when used for immunohistochemical staining of the tumor tissue. The additional predictive value of the signature markers compared to clinical parameters was 9% for patients with and 6% for patients without adjuvant therapy (as the best combination of clinical parameters only showed a predictive accuracy of 61% and 59%, respectively). Whereas the use of microarrays in clinical practice is limited by the large number of genes, complicated analytical methods, and the need for fresh-frozen tissue, RT-PCR or immunohistochemistry of a small number of proteins can be done routinely in a clinical setting.

Note that the samples were obtained during initial surgery, before any of the patients received adjuvant therapy. The expression signatures we identified predicted clinical outcomes specific for patients with and without adjuvant therapy. These signatures could be used to stratify patients for adjuvant treatment of the disease: A patient that is classified as low risk (good prognosis) by the adjuvant therapy signature should receive adjuvant chemotherapy treatment, whereas a patient that is classified as low risk by the no adjuvant therapy signature might have a longer survival without chemotherapy.

Our signature genes can also help to stratify pancreatic cancer patients for new therapies. STAT3 was found to be the best single prognostic marker, with a high expression of STAT3 indicating a high risk. STAT3 inhibitors might therefore be promising therapeutic agents. It is known that a large percentage of pancreatic cancers feature aberrantly activated STAT3 [59]. Very recently, novel STAT3 phosphorylation inhibitors were demonstrated to suppress growth in pancreatic cancer cell lines [60].

For breast cancer, an FDA approved microarray-based test that uses the 70-gene signature by van't Veer et al. [3] to assess the metastatic risk in patients with node negative breast cancer is commercially available and can be utilized clinically. In a validation study on an independent data set of 307 node-negative breast cancer patients [23], the 70-gene test was shown to have a sensitivity of 90% and a specificity of 42%. The accuracy, however, resulted in 50%, which is equivalent to guessing. The reported ROC curve for predicting time to distant metastases shows the same area under curve of 68% as our signature for predicting prognosis in patients with adjuvant therapy (**Figure 4A**). Using an appropriate cut-off, our signature also shows a high sensitivity of 83% with a specificity of 45% (upper right corner of the ROC curve, **Figure S4A**). It therefore can be used reliably to identify a group of patients who seem to benefit from the adjuvant therapy.

Our study emphasizes the benefit of systematic network-based approaches that incorporate background knowledge for identifying biologically relevant marker genes. Correlations between gene expression levels and a clinical variable of interest can arise simply by chance, without any underlying biological cause, especially with few patient samples. One example for such a spurious correlation in our screening dataset is the *HBA1* gene, which encodes for hemoglobin alpha, and which showed a strong negative correlation with survival. Although *HBA1* would have been a candidate marker when ranked merely by correlation, it was not ranked among the top ten markers by NetRank. Since we found the idea of a cancer tissue expressing hemoglobin interesting and worth exploring further, we decided to include HBA1 in the immunohistochemistry validation. However, immunohistochemical staining for hemoglobin in the validation dataset was incapable of defining significantly different risk groups. In addition, adding HBA1 to our signatures did not improve, but impaired their predictive accuracies. We conclude that the strong negative correlation of *HBA1* expression levels with survival time in the screening dataset might have been caused by chance and not by any underlying biologically relevant causality. Network-based methods such as NetRank can add such causality for example in the form of known gene regulatory networks, resulting in the identification of markers that are more likely to be truly relevant.

Recent work by the Ideker lab also emphasized the benefit of network-based approaches [61]. Their study demonstrated that markers based on protein interaction subnetworks are first more reproducible than individual marker genes and second can improve classification accuracy for the van't Veer breast cancer dataset by 8% compared to the original 70 genes [3]. A further approach documenting the usefulness of networks employed PageRank to identify genes cross-talking between already published cancer genes [62].

During the progress of our study, another study [63] was published which used PageRank on protein interaction networks to improve recursive feature elimination for support vector machine learning. They found that this improved the prediction of *ERBB2* status and relapse in breast cancer. However, neither of these two studies validated their genes on an external patient cohort to demonstrate the validity of markers found with PageRanking based on biological networks. Moreover, we found that the use of transcription factor–target networks yields more accurate signatures than the use of protein interactions networks in cancer outcome studies.

The use of background knowledge in order to get more robust and more biologically meaningful signatures comes at a price. It is in the nature of the NetRank algorithm to favor genes with many connections, since they can increase their ranking, whereas uncharacterized genes with no connections cannot. Hence, marker genes found with NetRank are more likely to be well known and well-described in the literature and less likely to be previously uncharacterized. We also found that the predictive accuracy of the immunohistochemistry -based markers was lower than that of the microarray-based markers. One potential bias stems from the different design of tissue microarrays, which vary in the number of cores per case, core size, and density. In addition, the semi-quantitative evaluation of the immunohistochemical staining tends to be less accurate and less objective than microarray-based gene expression profiling.

In conclusion, the expression signatures we identified predicted clinical outcomes in patients with surgically resected pancreatic ductal adenocarcinoma specific for patients with and without adjuvant therapy. Since these signatures could be used to stratify patients for adjuvant treatment of the disease, they are a potential additional piece of information in clinical decision making and can help to reduce costs, improve patient survival, and quality of life.

## Materials and Methods

### Screening dataset

Two hundred forty-four freshly frozen tissue samples of pancreatic adenocarcinoma were obtained from surgical specimens from patients who underwent operations between 1996 and 2007 at German university hospitals in Berlin, Dresden, Heidelberg, Mannheim, Munich, and Regensburg. Informed consent was obtained from all patients included in this study. From each of the frozen tissue samples, 4 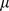
m slides were obtained, stained with hematoxylin and eosin, and re-evaluated by a pathologist (G. K.) experienced in pancreato-biliary pathology. Of these, 56 tissue samples contained tumorous tissue without any contamination from normal acini or islets and had suitable RNA quality. Of these, 30 were obtained from patients without any adjuvant therapy, and were used as the screening dataset. The clinical characteristics of this dataset are given in **Table 1**.

#### RNA preparation and array hybridization of the screening dataset samples

Total RNA was prepared from 10 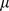
m thick sections using the RNeasy Mini Kit (Qiagen, Hilden, Germany) according to the manufacturer's recommendations. The quality of the prepared RNA was controlled using the Agilent RNA 6000 Pico Kit (Agilent, Böblingen, Germany). Only RNA samples with more than 3 ng/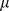
l RNA and an RNA integrity number greater than 4 were subjected to further analysis. From each sample 100 ng was used for cDNA synthesis and *in vitro* transcription-based amplification according to the Affymetrix GeneChip Two-Cycle Target Labeling and Control Kit (Affymetrix, Santa Clara, USA). Hybridization and detection of the labeled aRNA on the Affymetrix GeneChip Human Genome U133 Plus 2.0 (Affymetrix, Santa Clara, USA) were performed as recommended by the manufacturer.

#### Preprocessing and filtering of screening dataset microarray raw data

Affymetrix raw probe level intensity (CEL) files were background-corrected, normalized, and summarized using RMA [64]. The MIAME compliant data were deposited in the ArrayExpress database under the accession number E-MEXP-2780. Since our goal was to find a small number (five to ten) of reliable markers, we wanted to make sure that any candidate markers that we test further do not fall into one the categories of (i) low variance between all samples and therefore not discriminative, (ii) de facto not expressed, or (iii) expressed at very low levels and thus not reliably measured in our microarray data. This is achieved by the filtering steps described in the following. Note that for NetRank, filtering out meant actually not removing, but setting intial values to zero in order to prevent loss of edges from the network due to node removal.

First, to remove noise from genes with low expression, probe sets with a mean expression below 6 on the 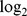 scale were filtered out from the dataset. Out of an initial 54,675 probe sets (measuring the expression of 20,111 genes), 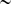
30,000 remained after this filtering. Second, genes whose expression shows little variation between patients are not informative as they cannot discriminate between patient groups. We therefore filtered out probe sets with a standard deviation below 0.5 on the 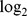 scale. This further reduced the number of probe sets to 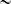
15,000. Third, we decided to keep for each gene only the probe set with the highest mean expression over all patients. We generally found a high correlation between probe sets reporting for the same gene. Keeping only the one probe set with the highest mean expression for each gene reduced the number of probe sets to 7,871 (measuring the expression of 7,871 genes). This is the size of the dataset that was used for all subsequent analyses. For NetRank, in order not to lose edges due to missing nodes, the size of the dataset was 20,111, with all except the 7,871 genes initialized with zero.

#### Quantitative real-time PCR (RT-PCR) of the screening dataset samples

RNA from the first amplification cycle was reverse transcribed into cDNA. One nanogram of cDNA was used for each TaqMan assay (Applied Biosystems, Weiterstadt, Germany). RT-PCR was performed using the TaqMan Universal PCR Master Mix (Applied Biosystems, Weiterstadt, Germany) according to the manufacturer's instructions. Gene expression of prognostic signature genes was quantified by the relative expression values using the following gene specific TaqMan Gene Expression Assays: BRCA1 (Hs01556193_m1), FOS (Hs01119266_g1), CDX2 (Hs01078080_m1), STAT3 (Hs00234174_m1), HBA2 (Hs00361191_g1), HBB (Hs00758889_s1), SRD5A1 (Hs00602694_mH), SP1 (Hs00293689_s1), JUN (Hs00277190_s1), USF1 (Hs00273038_m1), and CEBPA (Hs00269972_s1).

### Validation dataset

The validation dataset consisted of surgically resected PDAC samples from 517 patients who underwent operation between 1991 and 2008 at university hospitals in Berlin, Dresden, Jena, and Regensburg, Germany. Informed consent was obtained from all patients included in this study. Patients were followed up to 15 years by telephone inquiries, registry at cancer centers, and residents' registration offices. Out of the 517 patients, 105 were excluded because of missing data. The clinical characteristics of this dataset are given in **Table 1**. After the completion of this study we became aware of the fact that two patients (without adjuvant treatment) were present in both our screening and validation data set. To ensure that this caused no bias in our results, these two patients were excluded from all test sets in the validation analysis presented here.

#### Immunohistochemistry of the validation dataset

For immunohistochemical analysis, tissue microarrays with 1–4 cores per patient and with core sizes of 0.6 mm–2 mm were prepared using all the samples. The total number of cores was 1,696. Five 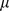
m thick sections were prepared using silanized slides (Menzel Gläser, Braunschweig, Germany). Immunohistochemistry was performed using the Benchmark System (Ventana, Illkirch, France) with the following antibodies and protocols: anti CDX2 (030044, DCS Innovative Diagnostik-Systeme, Hamburg, Germany, dilution 1∶30, UltraView DAB, CC1), anti c-Fos (sc-253, Santa Cruz Biotechnology, dilution 1∶100, pretreatment with heat), anti SP1 (sc-14027, Santa Cruz Biotechnology, dilution 1∶100, pretreatment with heat), anti CEBPA (sc-61, Santa Cruz Biotechnology, dilution 1∶100, pretreatment with heat), anti STAT3 (9139, Cell Signaling Technology, Frankfurt, Germany, dilution 1∶400, UltraView DAB, CC1), anti Jun (ab31367, Abcam, Cambridge, UK, dilution 1∶100, UltraView DAB, CC1), and anti BRCA1 (Calbiochem, dilution 1∶30, pretreatment with heat and enzymes). Afterwards the slides were briefly counterstained with hematoxylin. The staining was evaluated semi-quantitatively by a pathologist (G.K., D.A., or T.K.) without knowledge of the histopathological or molecular data into either four grades (negative, faint, moderate, and strong) or into percentages (0–100%) of strongly stained nuclei. For classification, the expression of each immunohistochemistry marker was dichotomized using the median value (median grade or median percentage) as a cut-off, resulting in two possible levels (low or high) for each marker.

### Classification procedures

Support vector machines are powerful supervised machine learning algorithms for classification problems [65]–[67]. We used a support vector machine to classify pancreatic tumors samples into poor or good prognosis groups based on the expression levels of selected genes. Here, we used the LIBSVM implementation as provided in the R package e1071 (version 1.5-18, obtained July 2008 from http://cran.r-project.org/web/packages/e1071/). The expression level of each gene was used as an independent feature to train the classifier. No kind of aggregation was used. All feature selection and machine learning steps were subjected to Monte Carlo cross-validation, which is a recommended and relatively un-biased evaluation strategy [22], [35] described in the following.

#### Monte Carlo cross-validation workflow

To get a robust estimate on the classification error rate, we adopted the multiple random validation strategy described by [22]. Given a fixed signature size 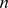 and a feature selection method, the following steps were repeated 1,000 times (see **Figure 1**):

1. The starting point is the screening dataset after filtering, consisting of a gene expression matrix with 7,871 features (rows) and 30 patient samples (columns). For NetRank, we additionally included genes (features) that did not pass filtering with their initial values set to zero.
2. The data are randomly split into training and test sets. The splitting is balanced such that the numbers of poor and good samples in the test set are either equal or differ by at most one. This is to ensure that there is no over-representation of one of the groups in the training set.
3. Using the training set data only, features are ranked according to a feature selection method (see below).
4. The top-ranked 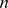 features are selected. These features become the signature.
5. The signature from the training set is used to train a classifier on the sample outcome, using the training set expression values of the signature genes as input.
6. The classifier is used to predict the outcome of the unseen test set patients.
7. The predicted outcome is compared with the true outcome. The fraction of correctly predicted patients defines the accuracy.

For NetRank, additional steps are taken between step 2 and step 3, which are explained the NetRank section below. The overall classification accuracy is the average of all repeated workflow accuracies. In order to ensure maximally comparable results, the random splits into training and test sets were carried out once and the sets were recorded. Thus, the exact same training and test sets were used for each method.

#### Calculation of prediction accuracy

Throughout the text, we use accuracy as the percentage of correctly classified samples, i. e. the sum of true positives and true negatives divided by the sum of true positives, false positives, true negatives, and false negatives.

#### Feature selection methods

Patients in the screening dataset were divided into two groups based on median survival time. The poor prognosis group consisted of patients who survived less than or equal to the median survival time, whereas the good prognosis group consisted of patients who survived longer than the median survival time. To select genes for a prognostic signature, the following methods for selecting genes were tested: (i) fold change, as defined by the ratio of mean gene expression in one group over the other group, (ii) the Student's t-statistic, (iii) the Pearson correlation coefficient of gene expression with survival time of the patient, and the Spearman rank correlation coefficient of gene expression with survival time of the patient, (iv) the SAM (Significance Analysis of Microarrays) method [34], and (v) our own NetRank algorithm. We also included selecting genes randomly.

### The NetRank algorithm for network-based gene ranking

For ranking of genes, NetRank combines the correlation of a gene's expression level with the survival time of the patient with a network of known gene–gene relationships. The ranking can be computed iteratively. Here, we follow the notation and implementation in [37]:
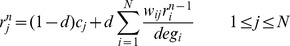
(1)Here 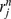 denotes the ranking of page 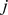 after 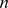 iterations, 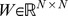 is a symmetric adjacency matrix for the gene network, so 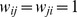 if genes 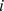 and 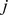 are connected, and 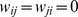 otherwise. 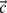 is a vector of absolute Pearson correlation coefficients of gene expression values with the patient survival time, and 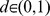 is a fixed parameter describing the influence of the network on the rank of a page. Setting 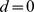 corresponds to no influence of the network and full influence of the gene expression data, whereas setting 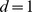 corresponds to full influence of the network and no influence of the gene expression data. The value 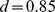 appears to be used by Google [26]. The rank of a gene depends on the rank of all genes that link to it. Scaling by 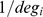 in the summation ensures that each gene has equal influence in the voting procedure. Each gene gets a rank of 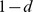 automatically and also gets 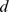 times the votes given by other genes.

The iteration to convergence in (1) corresponds to solving the equation
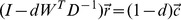
(2)where 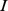 is the identity matrix, 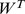 is the transpose of 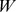, and 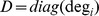. With the choice of 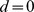 (no influence of the network, full influence of the gene expression data), equation (2) has the solution 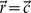. That is, the rank of a gene solely depends on the correlation of its expression with survival time. For 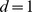 (full influence of the network, no influence of the gene expression data), equation (2) becomes
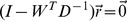
(3)


#### Monte Carlo cross-validation workflow for NetRank

The parameter 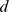 is set as part of the Monte Carlo cross-validation workflow. For NetRank, we added an additional inner cross-validation loop (see **Figure 1**, steps 2a to 2d). In this inner cross-validation, a part of the training set samples were set aside, and different values of 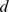 ranging from 0 to 1 in steps of 0.1 were used to run NetRank on the remaining training set samples. Accuracies of the top-ranked genes were then tested on the samples previously set aside. As a result of the inner cross-validation, one 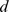 was chosen and used once for deriving a signature based on the whole training set, and evaluating its accuracy on the test set. It is important to note that no information of the test set is used for selecting 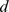, so the choice of a value for 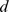 in the inner cross-validation does not rely on any prediction accuracy in the test set data. We found that a value of 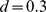 on the TRANSFAC network of transcription factors and their regulated targets gave the best results in terms of predictive accuracy of the identified signature genes. R code is available from the authors upon request.

#### Network datasets

For NetRank, results were based the TRANSFAC network which is defined by all human transcription factor–target pairs as provided in the TRANSFAC Suite 2008 [38]. Further networks tested were the HPRD network where genes are connected if there is a known interaction of their proteins in the Protein Reference Database (HPRD) [39], and the COXPRESdb network where genes are connected if their co-expression correlation coefficient as reported in the in the COXPRESdb database [40] is above 0.25. COXPRESdb contains co-expression values for all pairs of 19,777 human genes derived from a wide number of publicly available microarray datasets.

### Literature-based markers

To identify genes mentioned in the literature as prognostic immunohistochemistry markers for pancreatic cancer, we used GoGene [68] and combined the results of queries “pancrea* prognos* immunohisto* paraffin” and “pancrea* survival immunohisto* paraffin”. GoGene performs a PubMed query with the search term and then identifies gene names in the abstracts reported by PubMed. **Table S2** shows the literature genes with the PubMed IDs of the abstracts in which they were found.

## Supporting Information

Figure S1 **Survival time distribution in ten published cancer outcome studies.** (**A**)–(**J**) Histograms of survival times from ten published studies using microarray data from cancer patients for outcome prediction. For studies which define two prognosis groups, these groups are indicated by color (red, poor prognosis and blue, good prognosis). The dashed vertical line indicates the median.
(PDF)

Figure S2
**Examples of immunohistochemical staining of the marker candidates.** Antibody staining intensities were scored semi-quantitatively by a pathologist using four grades of negative (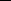), faint (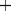), moderate (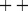), and strong (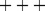) staining.(PDF)

Figure S3
**Survival by adjuvant therapy.** Out of 412 patients in the validation dataset, 172 patients who received adjuvant therapy had a lower 5-year-survival than the 240 patients who did not receive adjuvant therapy, although the difference is not significant (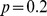, logrank test).(PDF)

Figure S4 **Receiver operating characteristic curves of signatures to predict risk.** (**A**) Signature to predict risk in patients with adjuvant therapy. The signature was developed with patients receiving adjuvant therapy separated by their median survival into two groups, a high risk group with shorter survival and a low risk group with longer survival. The signature consisted of the marker proteins STAT3, FOS, JUN, CDX2, CEBPA, and BRCA1. The receiver operating characteristic (ROC) curve of a classifier trained with this signature shows an area under the curve of 68% using leave-one-out cross-validation. (**B**) Signature to predict risk in patients without adjuvant therapy. The signature was developed with patients not receiving adjuvant therapy separated by their median survival into two groups, a high risk group with shorter survival and a low risk group with longer survival. The signature consisted of the marker proteins STAT3, JUN, SP1, CDX2, and BRCA1. The ROC curve of a classifier trained with this signature shows an area under the curve of 59% using leave-one-out cross-validation.
(PDF)

Figure S5 **Comparison of NetRank with a direct neighbor algorithm.** The plot shows the accuracy of a direct neighbor approach that only takes direct neighbors into account (as opposed to NetRank, which considers all nodes in the network) on the TRANSFAC network with different training set sizes. The direct neighbor approach performs almost identically to the Pearson correlation method (shown here for comparison). See below for a description of the direct neighbor method.
(PDF)

Figure S6 **Distribution of expression levels and correlation with survival in four distinct subsets of the full screening dataset.** (**A**) Histogram (density) of gene expression levels. Our filtering keeps only the high expression, high variance genes (red curve). Sizes of the four subsets are shown in the upper right. (**B**) Histogram (density) of absolute Pearson correlation coefficients of gene expression levels with patient survival. Since the red and the blue curve have very similar distribution, ranking by correlation (which is the starting point for our NetRank algorithm) will allow selection of uninformative, low variance genes (blue curve) that will impair prediction accuracy when included in a classifier. Hence, it is important to filter such genes out.
(PDF)

Table S1 **Microarray-based cancer classification studies on finding predictive signatures published in high-impact journals.** The studies were published in *Science*, *Nature*, *Nature Medicine*, *PNAS*, *PLoS Medicine*, *Cancer Cell*, *Lancet*, or *New England Journal of Medicine*. Some studies exhibit considerable flaws in methodology, as pointed out in the notes at the table bottom.
(PDF)

Table S2 **Fifty-one immunohistochemistry markers prognostic for survival in pancreatic cancer, found with a literature search.**
(PDF)

Table S3 **KEGG pathways most affected by signature genes and their interaction partners.**
(PDF)

Table S4 **Accuracies and standard errors for Monte Carlo cross-validations.** These numbers are the basis for the plots shown in Figure 2 and Figure S5.
(XLS)
