# Supplementary material for: Google Goes Cancer: Improving Outcome Prediction for Cancer Patients by Network-Based Ranking of Marker Genes
Source: PLoS Comput Biol. 2012 May 17;8(5):e1002511. doi: 10.1371/journal.pcbi.1002511 (PMC3355064; doi:10.1371/journal.pcbi.1002511)
Supplement: Figure S1 — Survival time distribution in ten published cancer outcome studies. (A)–(J) Histograms of survival times from ten published studies using microarray data from cancer patients for outcome prediction. For studies which define two prognosis groups, these groups are indicated by color (red, poor prognosis and blue, good prognosis). The dashed vertical line indicates the median. (PDF) [file pcbi.1002511.s001.pdf]

Figure S1. Survival time distribution in ten published cancer outcome studies. (**A**) – (**J**) Histograms of survival times from ten published studies using microarray data from cancer patients for outcome prediction. For studies which define two prognosis groups, these groups are indicated by color (red, poor prognosis and blue, good prognosis). The dashed vertical line indicates the median.

A

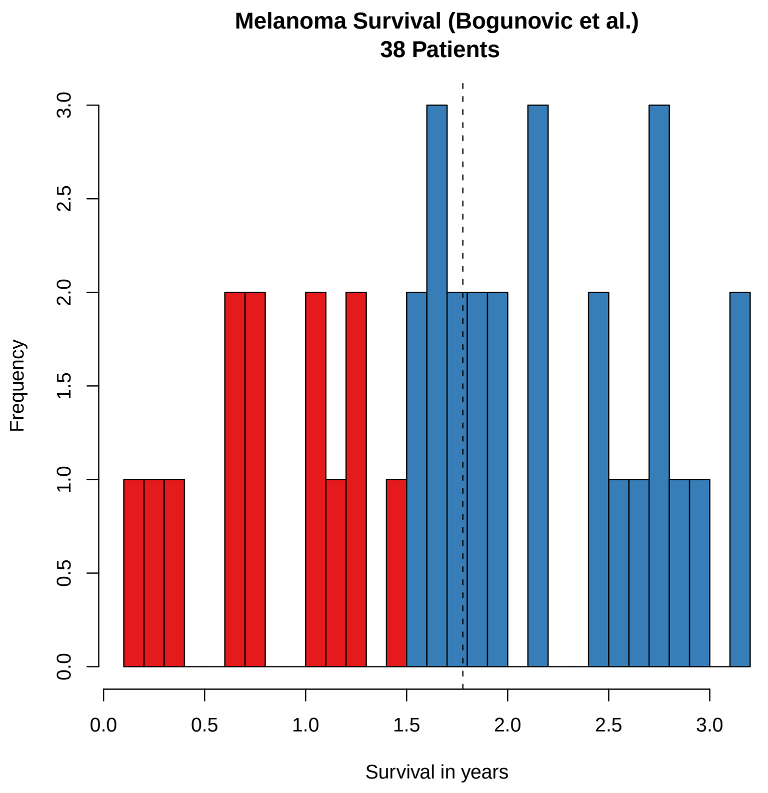

B

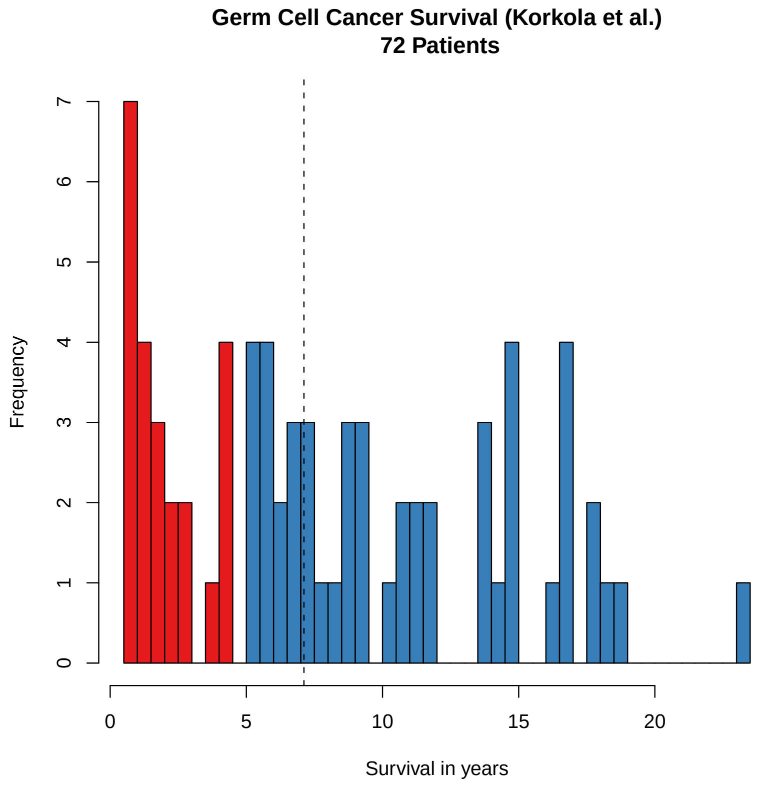

C

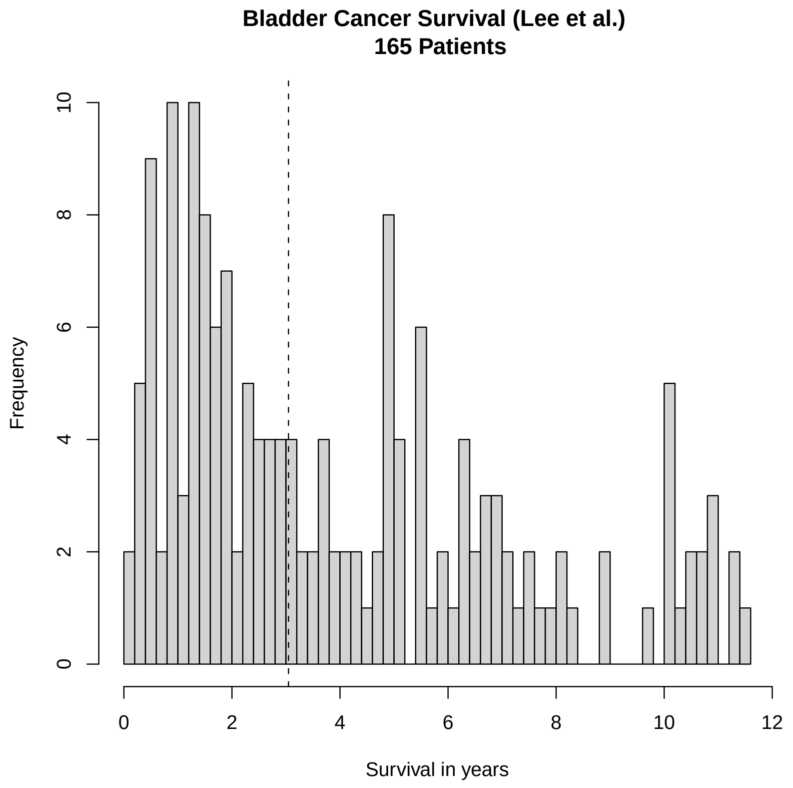
Bladder Cancer Survival (Lee et al.)
165 Patients
Frequency
0
2
4
6
8
10
0
2
4
6
8
10
12
Survival in years

D

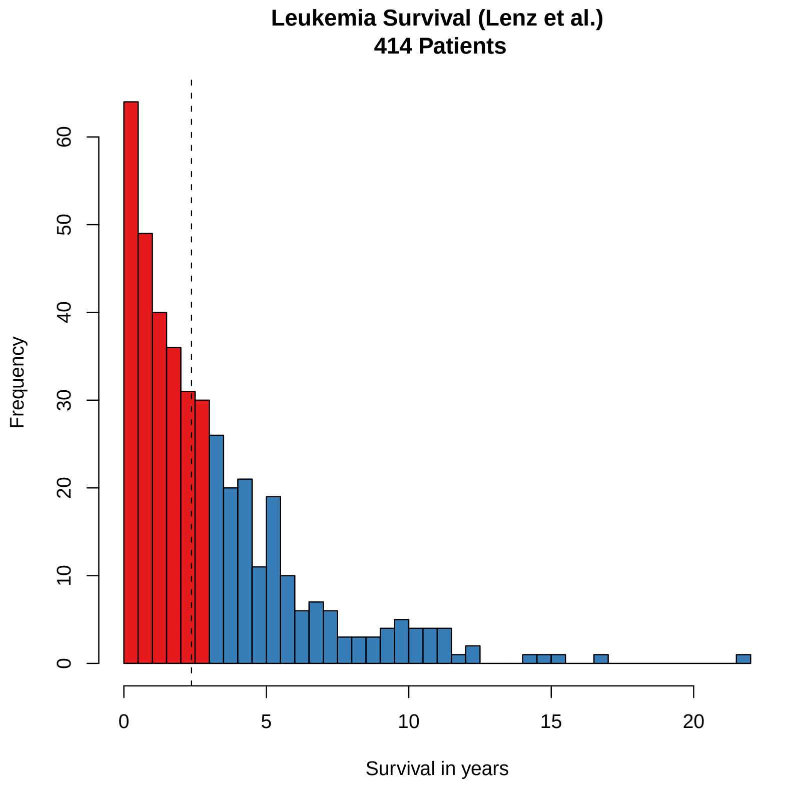
Leukemia Survival (Lenz et al.)
414 Patients
Frequency
0
10
20
30
40
50
60
0
5
10
15
20
Survival in years

E

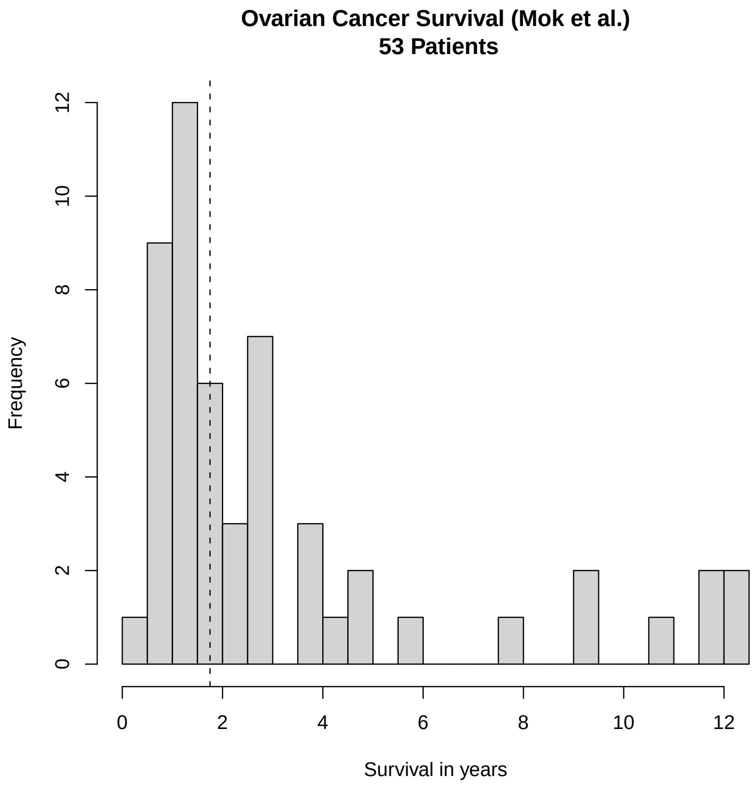
Ovarian Cancer Survival (Mok et al.)
53 Patients
Frequency
0
2
4
6
8
10
12
Survival in years

F

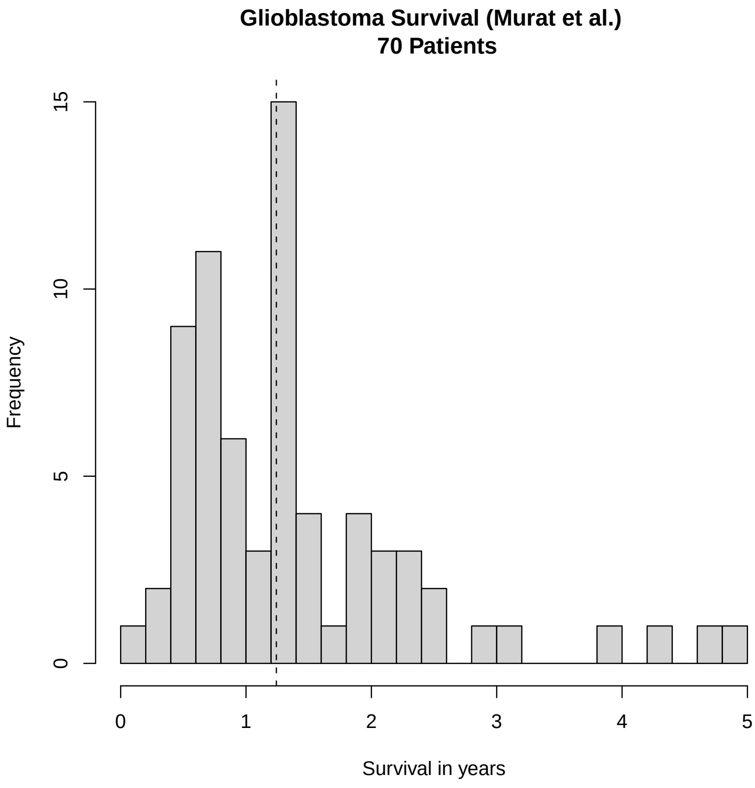
Glioblastoma Survival (Murat et al.)
70 Patients
Frequency
0
5
10
15
0
1
2
3
4
5
Survival in years

G

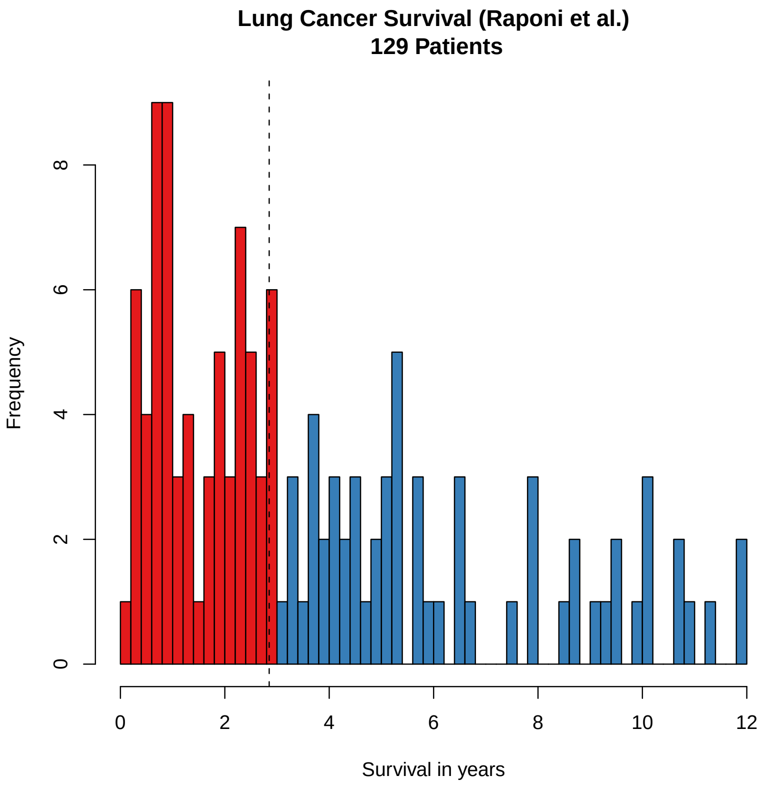
Lung Cancer Survival (Raponi et al.)
129 Patients
Frequency
0
2
4
6
8
0
2
4
6
8
10
12
Survival in years

H

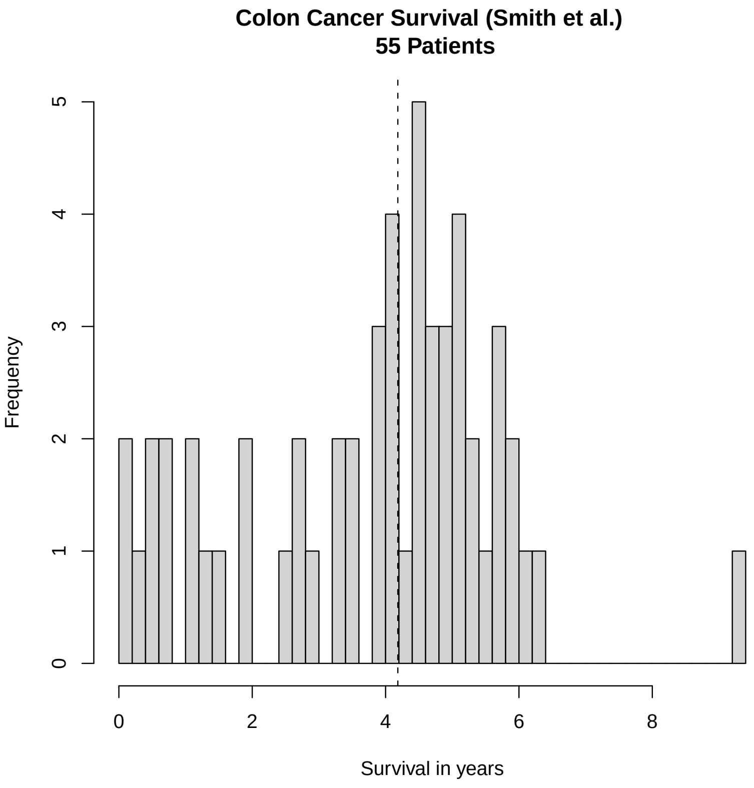
Colon Cancer Survival (Smith et al.)
55 Patients
Frequency
0
1
2
3
4
5
0
2
4
6
8
Survival in years

I

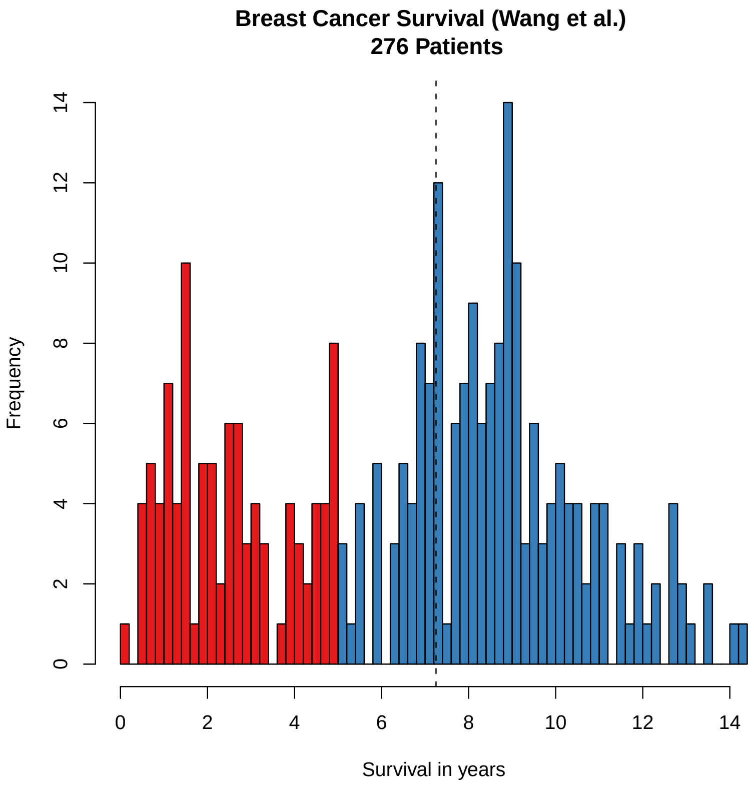
Breast Cancer Survival (Wang et al.)
276 Patients
Frequency
0
2
4
6
8
10
12
14
Survival in years

J

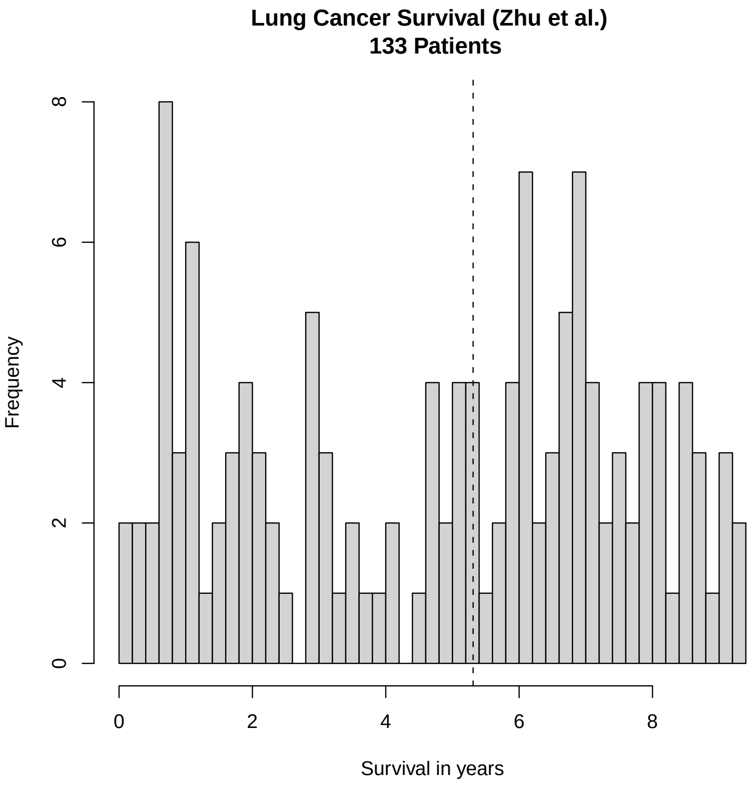
Lung Cancer Survival (Zhu et al.)
133 Patients
Frequency
0
2
4
6
8
Survival in years

References

Bogunovic D, O'Neill DW, Belitskaya-Levy I, Vacic V, Yu YL, Adams S, Darvishian F, Berman R, Shapiro R, Pavlick AC, et al. (2009) Immune profile and mitotic index of metastatic melanoma lesions enhance clinical staging in predicting patient survival. *Proc Natl Acad Sci U S A* **106**: 20429–34.

Korkola JE, Houldsworth J, Feldman DR, Olshen AB, Qin LX, Patil S, Reuter VE, Bosl GJ, and Chaganti RSK. (2009) Identification and validation of a gene expression signature that predicts outcome in adult men with germ cell tumors. *J Clin Oncol* **27**: 5240–7.

Lee JS, Leem SH, Lee SY, Kim SC, Park ES, Kim SB, Kim SK, Kim YJ, Kim WJ, and Chu IS. (2010) Expression signature of E2F1 and its associated genes predict superficial to invasive progression of bladder tumors. *J Clin Oncol* **28**: 2660–7.

Lenz G, Wright G, Dave SS, Xiao W, Powell J, Zhao H, Xu W, Tan B, Goldschmidt N, Iqbal J, et al. (2008) Stromal gene signatures in large-B-cell lymphomas. *N Engl J Med* **359**: 2313–23.

Mok SC, Bonome T, Vathipadiekal V, Bell A, Johnson ME, Wong Kk, Park DC, Hao K, Yip DKP, Donninger H, et al. (2009) A gene signature predictive for outcome in advanced ovarian cancer identifies a survival factor: microfibril-associated glycoprotein 2. *Cancer Cell* **16**: 521–32.

Murat A, Migliavacca E, Gorlia T, Lambiv WL, Shay T, Hamou MF, De Tribolet N, Regli L, Wick W, Kouwenhoven MCM, et al. (2008) Stem cell-related "self-renewal" signature and high epidermal growth factor receptor expression associated with resistance to concomitant chemoradiotherapy in glioblastoma. *J Clin Oncol* **26**: 3015–24.

Raponi M, Zhang Y, Yu J, Chen G, Lee G, Taylor JMG, Macdonald J, Thomas D, Moskaluk C, Wang Y, et al. (2006) Gene expression signatures for predicting prognosis of squamous cell and adenocarcinomas of the lung. *Cancer Res* **66**: 7466–72.

Smith JJ, Deane NG, Wu F, Merchant NB, Zhang B, Jiang A, Lu P, Johnson JC, Schmidt C, Bailey CE, et al. (2010) Experimentally derived metastasis gene expression profile predicts recurrence and death in patients with colon cancer. *Gastroenterology* **138**: 958–68.

Wang Y, Klijn JGM, Zhang Y, Sieuwerts AM, Look MP, Yang F, Talantov D, Timmermans M, Meijer-van Gelder ME, Yu J, et al. (2005) Gene-expression profiles to predict distant metastasis of lymph-node-negative primary breast cancer. *Lancet* **365**: 671–9.

Zhu CQ, Ding K, Strumpf D, Weir BA, Meyerson M, Pennell N, Thomas RK, Naoki K, Ladd-Acosta C, Liu N, et al. (2010) Prognostic and predictive gene signature for adjuvant chemotherapy in resected non-small-cell lung cancer. *J Clin Oncol* **28**: 4417–24.
