# Supplementary figures and images for: Google Goes Cancer: Improving Outcome Prediction for Cancer Patients by Network-Based Ranking of Marker Genes
Source: PLoS Comput Biol. 2012 May 17;8(5):e1002511. doi: 10.1371/journal.pcbi.1002511 (PMC3355064; doi:10.1371/journal.pcbi.1002511)

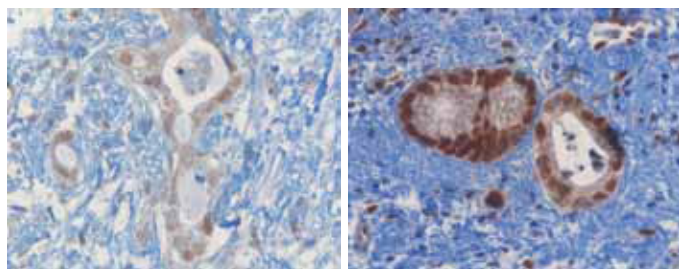

**+**      **FOS**      **+++**

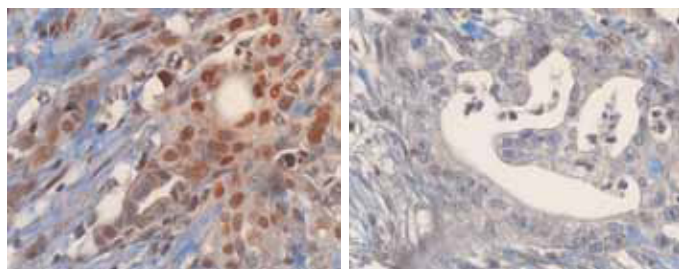

**+++**      **JUN**      **-**

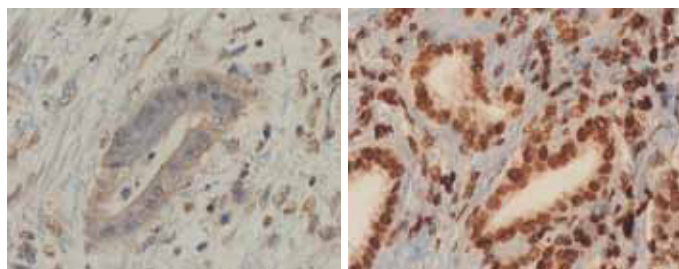

**-**      **BRCA1**      **+++**

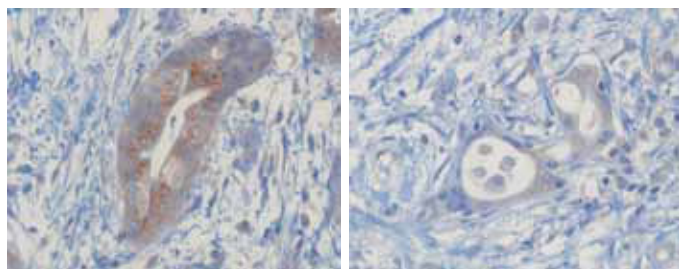

**++**      **CEBPA**      **-**

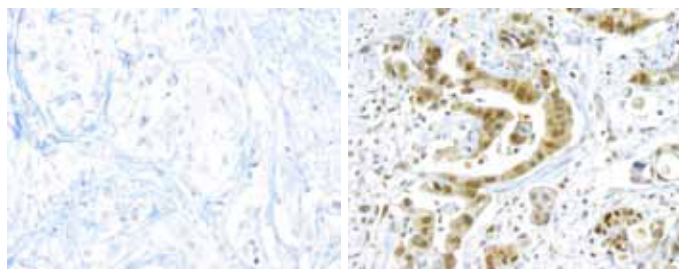

**-**      **STAT3**      **+++**

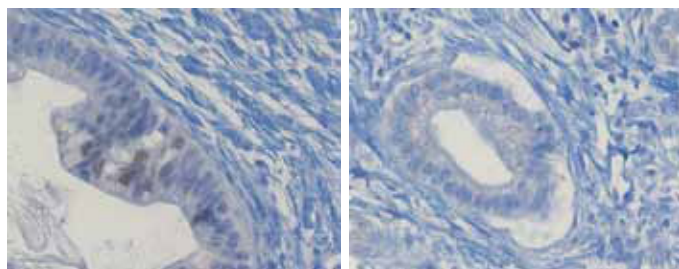

**++**      **CDX2**      **-**

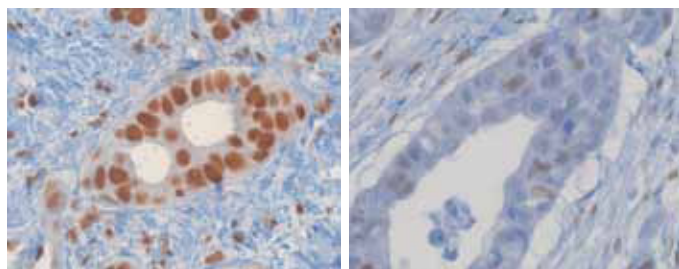

**+++**      **SP1**      **+**

Supplement: Figure S2 — Examples of immunohistochemical staining of the marker candidates. Antibody staining intensities were scored semi-quantitatively by a pathologist using four grades of negative (), faint (), moderate (), and strong () staining. (PDF) [file pcbi.1002511.s002.pdf]

## Survival by adjuvant therapy

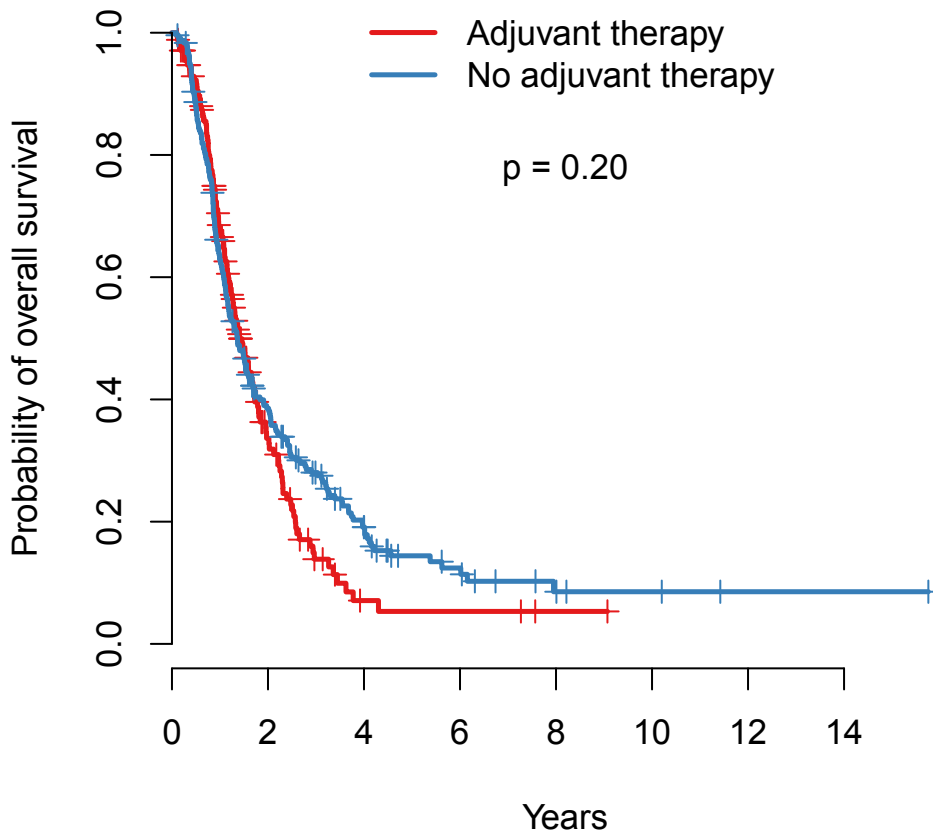

No. at risk

|                     |     |    |    |    |   |   |   |   |
|---------------------|-----|----|----|----|---|---|---|---|
| Adjuvant therapy    | 172 | 38 | 4  | 3  | 1 |   |   |   |
| No adjuvant therapy | 240 | 83 | 32 | 12 | 5 | 3 | 1 | 1 |

Supplement: Figure S3 — Survival by adjuvant therapy. Out of 412 patients in the validation dataset, 172 patients who received adjuvant therapy had a lower 5-year-survival than the 240 patients who did not receive adjuvant therapy, although the difference is not significant (, logrank test). (PDF) [file pcbi.1002511.s003.pdf]
