# Supplementary material for: Google Goes Cancer: Improving Outcome Prediction for Cancer Patients by Network-Based Ranking of Marker Genes
Source: PLoS Comput Biol. 2012 May 17;8(5):e1002511. doi: 10.1371/journal.pcbi.1002511 (PMC3355064; doi:10.1371/journal.pcbi.1002511)
Supplement: Figure S4 — Receiver operating characteristic curves of signatures to predict risk. (A) Signature to predict risk in patients with adjuvant therapy. The signature was developed with patients receiving adjuvant therapy separated by their median survival into two groups, a high risk group with shorter survival and a low risk group with longer survival. The signature consisted of the marker proteins STAT3, FOS, JUN, CDX2, CEBPA, and BRCA1. The receiver operating characteristic (ROC) curve of a classifier trained with this signature shows an area under the curve of 68% using leave-one-out cross-validation. (B) Signature to predict risk in patients without adjuvant therapy. The signature was developed with patients not receiving adjuvant therapy separated by their median survival into two groups, a high risk group with shorter survival and a low risk group with longer survival. The signature consisted of the marker proteins STAT3, JUN, SP1, CDX2, and BRCA1. The ROC curve of a classifier trained with this signature shows an area under the curve of 59% using leave-one-out cross-validation. (PDF) [file pcbi.1002511.s004.pdf]

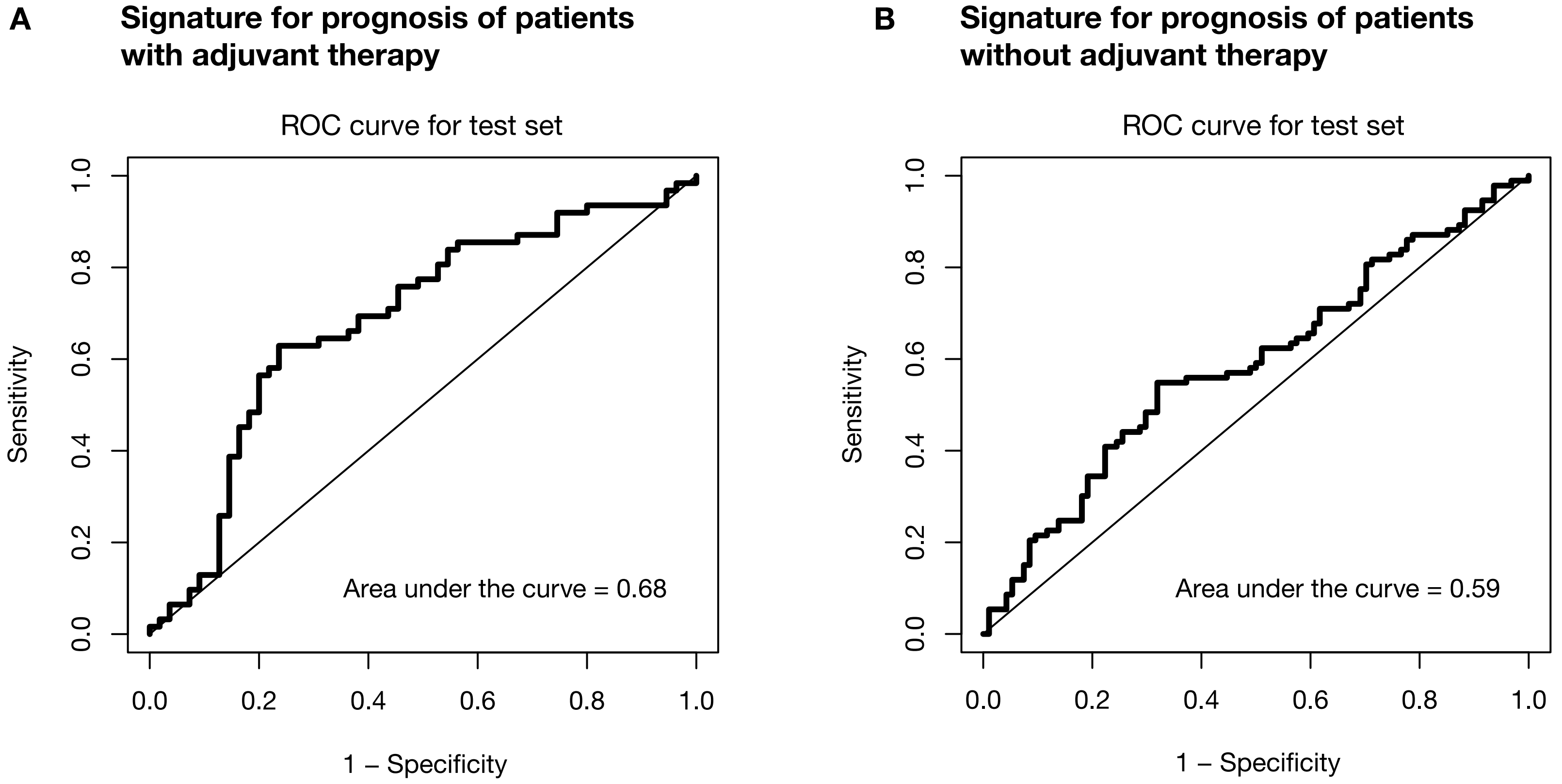
A
Signature for prognosis of patients with adjuvant therapy
ROC curve for test set
Sensitivity
1 – Specificity
Area under the curve = 0.68
0.0
0.2
0.4
0.6
0.8
1.0
B
Signature for prognosis of patients without adjuvant therapy
ROC curve for test set
Sensitivity
1 – Specificity
Area under the curve = 0.59
0.0
0.2
0.4
0.6
0.8
1.0
