# Supplementary material for: Google Goes Cancer: Improving Outcome Prediction for Cancer Patients by Network-Based Ranking of Marker Genes
Source: PLoS Comput Biol. 2012 May 17;8(5):e1002511. doi: 10.1371/journal.pcbi.1002511 (PMC3355064; doi:10.1371/journal.pcbi.1002511)
Supplement: Figure S5 — Comparison of NetRank with a direct neighbor algorithm. The plot shows the accuracy of a direct neighbor approach that only takes direct neighbors into account (as opposed to NetRank, which considers all nodes in the network) on the TRANSFAC network with different training set sizes. The direct neighbor approach performs almost identically to the Pearson correlation method (shown here for comparison). See below for a description of the direct neighbor method. (PDF) [file pcbi.1002511.s005.pdf]

Figure S5. Comparison of NetRank with a direct neighbor algorithm. The plot shows the accuracy of a direct neighbor approach that only takes direct neighbors into account (as opposed to NetRank, which considers all nodes in the network) on the TRANSFAC network with different training set sizes. The direct neighbor approach performs almost identically to the Pearson correlation method (shown here for comparison). See below for a description of the direct neighbor method.

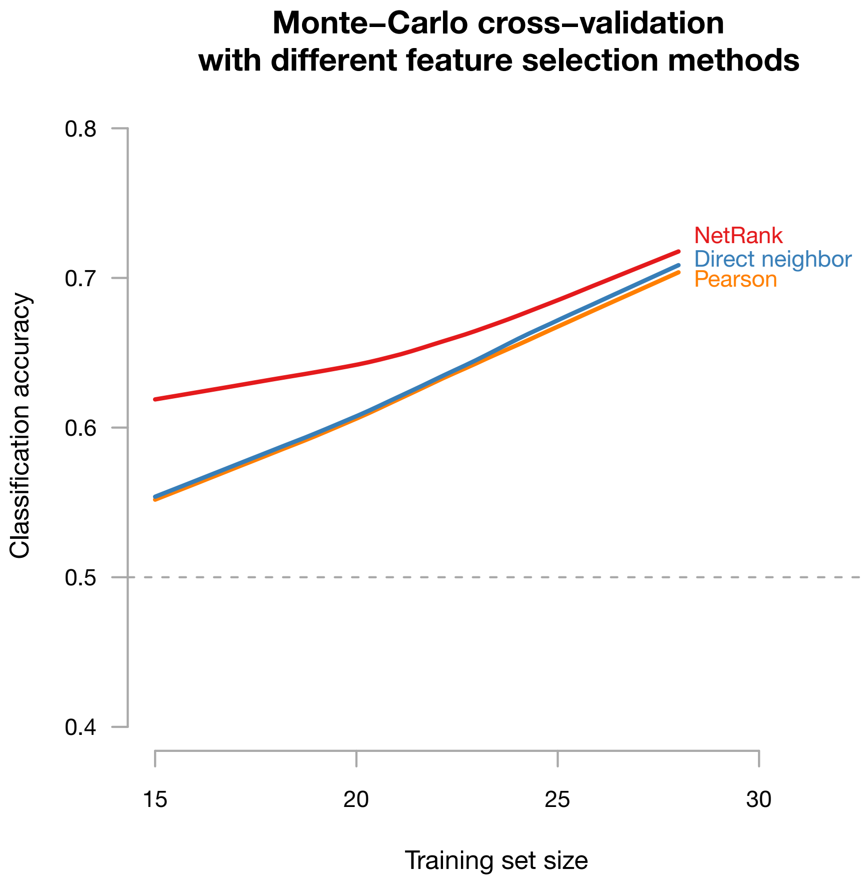

Direct neighbor method

The direct neighbor method averages a gene's correlation coefficient over its direct neighbors. Similar to NetRank, the direct neighbor method starts with an undirected gene network W (represented by a symmetric adjacency matrix with $w_{ij} = w_{ji} = 1$ if genes i and j are connected, and $w_{ij} = w_{ji} = 0$ otherwise) and a gene vector $\vec{c}$ with absolute Pearson correlation coefficients of gene expression values with the patient survival time.

The rank r_i of a gene i is then determined by

$$\vec{r} = (W\vec{c})/\vec{deg}_W \tag{1}$$

where $\vec{deg}_W$ denotes the degrees (column sums) of the matrix W.
