# Supplementary material for: Google Goes Cancer: Improving Outcome Prediction for Cancer Patients by Network-Based Ranking of Marker Genes
Source: PLoS Comput Biol. 2012 May 17;8(5):e1002511. doi: 10.1371/journal.pcbi.1002511 (PMC3355064; doi:10.1371/journal.pcbi.1002511)
Supplement: Figure S6 — Distribution of expression levels and correlation with survival in four distinct subsets of the full screening dataset. (A) Histogram (density) of gene expression levels. Our filtering keeps only the high expression, high variance genes (red curve). Sizes of the four subsets are shown in the upper right. (B) Histogram (density) of absolute Pearson correlation coefficients of gene expression levels with patient survival. Since the red and the blue curve have very similar distribution, ranking by correlation (which is the starting point for our NetRank algorithm) will allow selection of uninformative, low variance genes (blue curve) that will impair prediction accuracy when included in a classifier. Hence, it is important to filter such genes out. (PDF) [file pcbi.1002511.s006.pdf]

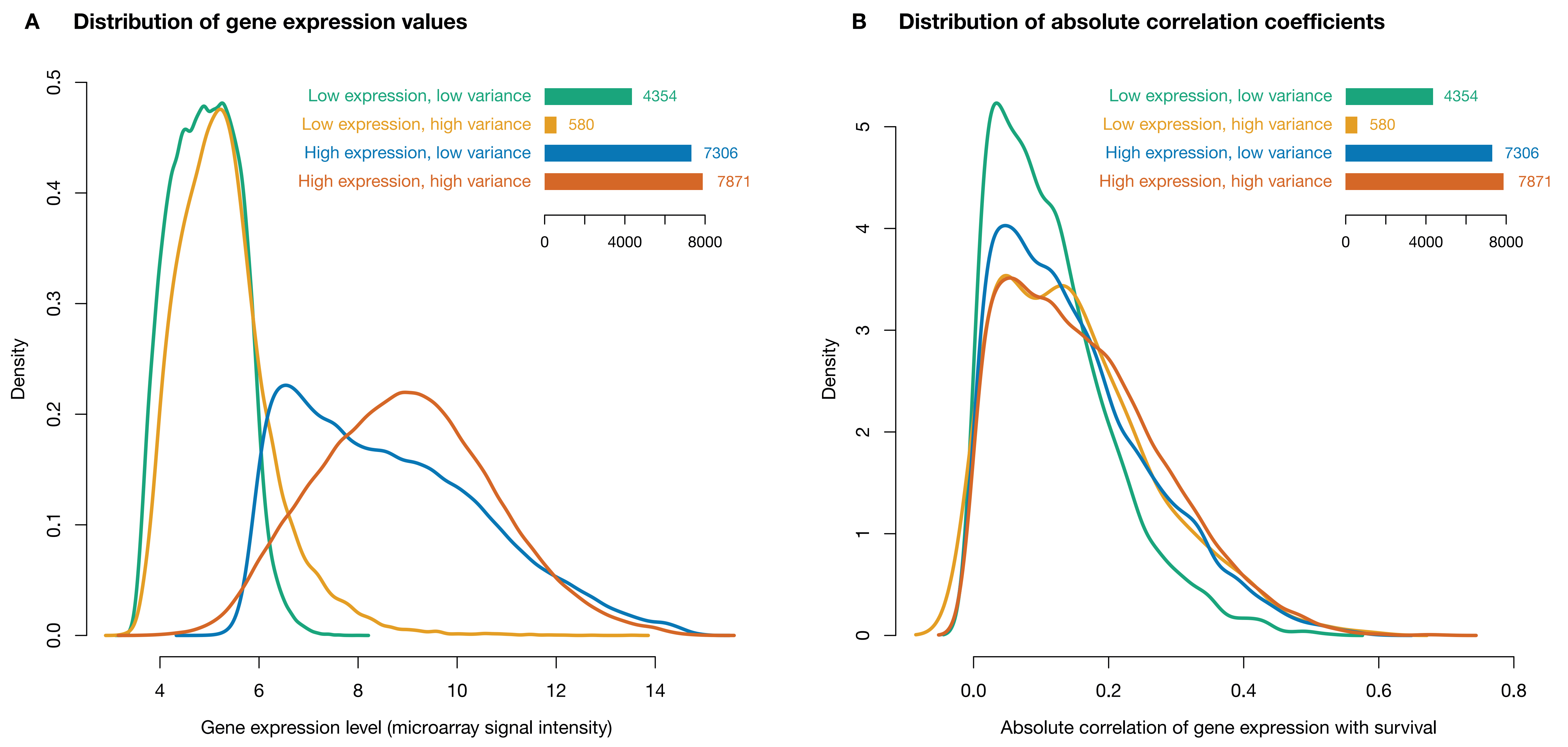

A
Distribution of gene expression values
Low expression, low variance
4354
Low expression, high variance
580
High expression, low variance
7306
High expression, high variance
7871
0
4000
8000
Density
0.0
0.1
0.2
0.3
0.4
0.5
4
6
8
10
12
14
Gene expression level (microarray signal intensity)
B
Distribution of absolute correlation coefficients
Low expression, low variance
4354
Low expression, high variance
580
High expression, low variance
7306
High expression, high variance
7871
0
4000
8000
Density
0
1
2
3
4
5
0.0
0.2
0.4
0.6
0.8
Absolute correlation of gene expression with survival
