# Supplementary material for: Google Goes Cancer: Improving Outcome Prediction for Cancer Patients by Network-Based Ranking of Marker Genes
Source: PLoS Comput Biol. 2012 May 17;8(5):e1002511. doi: 10.1371/journal.pcbi.1002511 (PMC3355064; doi:10.1371/journal.pcbi.1002511)
Supplement: Table S1 — Microarray-based cancer classification studies on finding predictive signatures published in high-impact journals. The studies were published in Science, Nature, Nature Medicine, PNAS, PLoS Medicine, Cancer Cell, Lancet, or New England Journal of Medicine. Some studies exhibit considerable flaws in methodology, as pointed out in the notes at the table bottom. (PDF) [file pcbi.1002511.s007.pdf]

Table S1. Microarray-based cancer classification studies on finding predictive signatures published in high-impact journals. The studies were published in *Science, Nature, Nature Medicine, PNAS, PLoS Medicine, Cancer Cell, Lancet,* or *New England Journal of Medicine.* Some studies exhibit considerable flaws in methodology, as pointed out in the notes at the table bottom.

Study	Disease	Number of patient samples	Number of genes in signature	Clinical parameter
Golub et al. (1999)	Acute myeloid leukemia vs. acute lymphoblastic leukemia	72	50	Disease classification
Alon et al. (1999)	Colon cancer	62	Not reported	Tumor vs. healthy tissue
Alizadeh et al. (2000)	Diffuse large-B-cell lymphoma	40	Not reported	Disease subclassification
Khan et al. (2001) [a, b]	Small round blue cell tumors	88	96	Disease classification
Sørlie et al. (2001)	Breast cancer	85	456, 264	Tumor subclasses, Survival
West et al. (2001)	Breast cancer	49	100	Outcome
Shipp et al. (2002)	Diffuse large B-cell lymphoma	77, 58	30, 13	Subclassification, Outcome
Rosenwald et al. (2002) [c, d]	Diffuse large B-cell lymphoma	240	17	Survival
Yeoh et al. (2002)	Acute lymphoblastic leukemia	327	7–20	Subclassification and outcome
Pomeroy et al. (2002) [e]	Medulloblastoma	60	8	Survival
Beer et al. (2002)	Lung adenocarcinoma	86	50	Survival
van 't Veer et al. (2002) [c, f]	Breast cancer	117	70	5-year metastasis-free survival
van de Vijver et al. (2002)	Breast cancer	295	70	Prognosis
Iizuka et al. (2003) [c]	Hepatocellular carcinoma	60	12	1-year recurrence-free survival
Huang et al. (2003) [g]	Breast cancer	89	Metagenes	Nodal metastatic states and relapse
Dave et al. (2004) [h]	Follicular lymphoma	191	67	Survival
Lossos et al. (2004)	Diffuse large B-cell lymphoma	66	6	Survival
Bullinger et al. (2004)	Acute myeloid leukemia	116	133	Survival
Wang et al. (2005)	Breast cancer	286	76	Distant metastasis
Dave et al. (2006)	Burkitt's lymphoma vs. diffuse large-B-cell lymphoma	303	217	Disease classification
Zhao et al. (2006)	Renal cell carcinoma	177	259	Survival
Shedden et al. (2008)	Lung adenocarcinoma	442	Various	Survival
Lenz et al. (2008)	Diffuse large-B-cell lymphoma	414	39, 283, 71	Survival after treatment
Boutros et al. (2009)	Non-small-cell lung cancer	147	6	Survival

a Tibshirani and Efron (2002) found that the complex neural network model used by the authors is essentially extracting linear principal components, and thus is unnecessarily complicated for this problem

b Lai et al. (2006) pointed out an information leak biasing the results caused by the authors' use of the complete dataset (including the validation set) for gene subset selection

c Michiels et al. (2005) found that the published misclassification rates were below the lower 95% confidence limit obtained by random validation

d Segal (2006) pointed out an information leak because test set data was used for an initial clustering

e Michiels et al. (2005) used a multiple random validation strategy on the same data, and found that the original study did not classify patients better than chance

f Tibshirani and Efron (2002) tried but were unable to exactly reproduce the analysis, even with help of the authors. Ein-Dor et al. (2005) found that the list of 70 genes found by the authors was highly unstable.

g Ruschhaupt et al. (2004) found only 75% accuracy instead of the 90% reported by the authors

h Tibshirani (2005) re-analyzed the data and found that the authors' results were extremely fragile—in particular, when their equal-sized training and test sets were swapped, the authors' finding disappeared and virtually nothing was significant

References

Alizadeh AA, Eisen MB, Davis RE, Ma C, Lossos IS, Rosenwald A, Boldrick JC, Sabet H, Tran T, Yu X, et al. (2000) Distinct types of diffuse large B-cell lymphoma identified by gene expression profiling. *Nature* **403**: 503–11.

Alon U, Barkai N, Notterman DA, Gish K, Ybarra S, Mack D, and Levine AJ. (1999) Broad patterns of gene expression revealed by clustering analysis of tumor and normal colon tissues probed by oligonucleotide arrays. *Proc Natl Acad Sci U S A* **96**: 6745–50.

Beer DG, Kardia SLR, Huang CC, Giordano TJ, Levin AM, Misek DE, Lin L, Chen G, Gharib TG, Thomas DG, et al. (2002) Gene-expression profiles predict survival of patients with lung adenocarcinoma. *Nat Med* **8**: 816–24.

Boutros PC, Lau SK, Pintilie M, Liu N, Shepherd FA, Der SD, Tsao MS, Penn LZ, and Jurisica I. (2009) Prognostic gene signatures for non-small-cell lung cancer. *Proc Natl Acad Sci U S A* **106**: 2824–8.

Bullinger L, Döhner K, Bair E, Fröhling S, Schlenk RF, Tibshirani R, Döhner H, and Pollack JR. (2004) Use of gene-expression profiling to identify prognostic subclasses in adult acute myeloid leukemia. *N Engl J Med* **350**: 1605–16.

Dave SS, Fu K, Wright GW, Lam LT, Kluin P, Boerma EJ, Greiner TC, Weisenburger DD, Rosenwald A, Ott G, et al. (2006) Molecular diagnosis of Burkitt's lymphoma. *N Engl J Med* **354**: 2431–2442.

Dave SS, Wright G, Tan B, Rosenwald A, Gascoyne RD, Chan WC, Fisher RI, Braziel RM, Rimsza LM, Grogan TM, et al. (2004) Prediction of survival in follicular lymphoma based on molecular features of tumor-infiltrating immune cells. *N Engl J Med* **351**: 2159–69.

Ein-Dor L, Kela I, Getz G, Givol D, and Domany E. (2005) Outcome signature genes in breast cancer: is there a unique set? *Bioinformatics* **21**: 171–178.

Golub TR, Slonim DK, Tamayo P, Huard C, Gaasenbeek M, Mesirov JP, Coller H, Loh ML, Downing JR, Caligiuri MA, et al. (1999) Molecular Classification of Cancer: Class Discovery and Class Prediction by Gene Expression Monitoring. *Science* **286**: 531–537.

Huang E, Cheng SH, Dressman H, Pittman J, Tsou MH, Horng CF, Bild A, Iversen ES, Liao M, Chen CM, et al. (2003) Gene expression predictors of breast cancer outcomes. *Lancet* **361**: 1590–1596.

Iizuka N, Oka M, Yamada-Okabe H, Nishida M, Maeda Y, Mori N, Takao T, Tamesa T, Tangoku A, Tabuchi H, et al. (2003) Oligonucleotide microarray for prediction of early intrahepatic recurrence of hepatocellular carcinoma after curative resection. *Lancet* **361**: 923–9.

Khan J, Wei JS, Ringnér M, Saal LH, Ladanyi M, Westermann F, Berthold F, Schwab M, Antonescu CR, Peterson C, et al. (2001) Classification and diagnostic prediction of cancers using gene expression profiling and artificial neural networks. *Nat Med* **7**: 673–9.

Lai C, Reinders MJT, Van't Veer LJ, and Wessels LFA. (2006) A comparison of univariate and multivariate gene selection techniques for classification of cancer datasets. *BMC Bioinformatics* **7**: 235.

Lenz G, Wright G, Dave SS, Xiao W, Powell J, Zhao H, Xu W, Tan B, Goldschmidt N, Iqbal J, et al. (2008) Stromal gene signatures in large-B-cell lymphomas. *N Engl J Med* **359**: 2313–23.

Lossos IS, Czerwinski DK, Alizadeh AA, Wechser MA, Tibshirani R, Botstein D, and Levy R. (2004) Prediction of survival in diffuse large-B-cell lymphoma based on the expression of six genes. *N Engl J Med* **350**: 1828–37.

Michiels S, Koscielny S, and Hill C. (2005) Prediction of cancer outcome with microarrays: a multiple random validation strategy. *Lancet* **365**: 488–492.

Pomeroy SL, Tamayo P, Gaasenbeek M, Sturla LM, Angelo M, McLaughlin ME, Kim JYH, Goumnerova LC, Black PM, Lau C, et al. (2002) Prediction of central nervous system embryonal tumour outcome based on gene expression. *Nature* **415**: 436–42.

Rosenwald A, Wright G, Chan WC, Connors JM, Campo E, Fisher RI, Gascoyne RD, Muller-Hermelink KH, Smeland EB, Giltnane JM, et al. (2002) The Use of Molecular Profiling to Predict Survival after Chemotherapy for Diffuse Large-B-Cell Lymphoma. *N Engl J Med* **346**: 1937–1947.

Ruschhaupt M, Huber W, Poustka A, and Mansmann U. (2004) A compendium to ensure computational reproducibility in high-dimensional classification tasks. *Stat Appl Genet Mol Biol* **3**: Article37.

Segal MR. (2006) Microarray gene expression data with linked survival phenotypes: diffuse large-B-cell lymphoma revisited. *Biostatistics* **7**: 268–85.

Shedden K, Taylor JMG, Enkemann SAA, Tsao MSS, Yeatman TJJ, Gerald WLL, Eschrich S, Jurisica I, Giordano TJJ, Misek DEE, et al. (2008) Gene expression-based survival prediction in lung adenocarcinoma: a multi-site, blinded validation study. *Nat Med* **14**: 822–827.

Shipp MA, Ross KN, Tamayo P, Weng AP, Kutok JL, Aguiar RC, Gaasenbeek M, Angelo M, Reich M, Pinkus GS, et al. (2002) Diffuse large B-cell lymphoma outcome prediction by gene-expression profiling and supervised machine learning. *Nat Med* **8**: 68–74.

Sørlie T, Perou CM, Tibshirani R, Aas T, Geisler S, Johnsen H, Hastie T, Eisen MB, De Rijn MvdR, Jeffrey SS, et al. (2001) Gene expression patterns of breast carcinomas distinguish tumor subclasses with clinical implications. *Proc Natl Acad Sci U S A* **98**: 10869–74.

Tibshirani RJ and Efron B. (2002) Pre-validation and inference in microarrays. *Stat Appl Genet Mol Biol* **1**: Article 1.

Tibshirani R (2005). *Re-analysis of Dave et al, NEJM Nov 18, 2004*. Tech. rep. `http://www-stat.stanford.edu/~tibs/FL/report`.

Van de Vijver MJ, He YD, Van't Veer LJ, Dai H, Hart AA, Voskuil DW, Schreiber GJ, Peterse JL, Roberts C, Marton MJ, et al. (2002) A gene-expression signature as a predictor of survival in breast cancer. *N Engl J Med* **347**: 1999–2009.

Van 't Veer LJ, Dai H, Van de Vijver MJ, He YD, Hart AA, Mao M, Peterse HL, Van der Kooy K, Marton MJ, Witteveen AT, et al. (2002) Gene expression profiling predicts clinical outcome of breast cancer. *Nature* **415**: 530–536.

Wang Y, Klijn JGM, Zhang Y, Sieuwerts AM, Look MP, Yang F, Talantov D, Timmermans M, Meijer-van Gelder ME, Yu J, et al. (2005) Gene-expression profiles to predict distant metastasis of lymph-node-negative primary breast cancer. *Lancet* **365**: 671–9.

West M, Blanchette C, Dressman H, Huang E, Ishida S, Spang R, Zuzan H, Olson JAJ, Marks JR, and Nevins JR. (2001) Predicting the clinical status of human breast cancer by using gene expression profiles. *Proc Natl Acad Sci U S A* **98**: 11462–7.

Yeoh EJ, Ross ME, Shurtleff SA, Williams WK, Patel D, Mahfouz R, Behm FG, Raimondi SC, Relling MV, Patel A, et al. (2002) Classification, subtype discovery, and prediction of outcome in pediatric acute lymphoblastic leukemia by gene expression profiling. *Cancer Cell* **1**: 133–43.

Zhao H, Ljungberg B, Grankvist K, Rasmuson T, Tibshirani R, and Brooks JD. (2006) Gene expression profiling predicts survival in conventional renal cell carcinoma. *PLoS Med* **3**: e13.
