# Supplementary material for: Google Goes Cancer: Improving Outcome Prediction for Cancer Patients by Network-Based Ranking of Marker Genes
Source: PLoS Comput Biol. 2012 May 17;8(5):e1002511. doi: 10.1371/journal.pcbi.1002511 (PMC3355064; doi:10.1371/journal.pcbi.1002511)
Supplement: Table S2 — Fifty-one immunohistochemistry markers prognostic for survival in pancreatic cancer, found with a literature search. (PDF) [file pcbi.1002511.s008.pdf]

Table S2. Fifty-one immunohistochemistry markers prognostic for survival in pancreatic cancer, found with a literature search.

Entrez Gene ID	Gene Symbol	PubMed IDs
1027	CDKN1B	16086840, 16528608, 16951171, 17035403, 18980972
1029	CDKN2A	17278096, 17420962, 18813127, 19200576, 19664933
10855	HPSE	11953884, 16110255, 16388520, 17715045
1508	CTSB	11185708, 15367886
1514	CTSL1	2297706, 8694562, 15367886
1647	GADD45A	12171884, 16274047, 17230496, 17940512
1890	TYMP	11745251, 12717266, 16137582, 17443278, 19760965
2810	SFN	15908786, 19047086
3068	HDGF	17062679
3091	HIF1A	18829975, 18927305, 19352387, 19372568, 19690016
332	BIRC5	16202147
3655	ITGA6	14707725
387	RHOA	18495213
3918	LAMC2	11920553
3958	LGALS3	11815981, 12171885, 12824888, 15548371, 16007065
4089	SMAD4	15832084, 16137582, 17203996, 18425078, 19165547
4102	MAGEA3	16331618
4312	MMP1	11181677, 12066203, 15714128, 16678855, 19887609
4313	MMP2	17120744, 17377415, 18630474, 19165547, 19924022
4316	MMP7	12066203, 15102692, 15810077, 16699793, 16858545
4478	MSN	15908786, 17874463
4583	MUC2	19236510, 19276352, 19347361, 19412570, 19587537
4585	MUC4	17079945, 17621592, 17676483, 18475301, 19236510
4586	MUC5AC	18475301, 19154264, 19276352, 19347361, 19587537
5176	SERPINF1	15150108
5243	ABCB1	9537202, 11039467, 15688370, 15841045, 19598259
5268	SERPINB5	15053038, 15842640, 17396143, 19389518, 19664933
5292	PIM1	18708761, 19528349
5320	PLA2G2A	19342650
5468	PPARG	11745416, 17085658, 19396032
5743	PTGS2	15532792, 16467169, 18059224, 19098866, 19389518
581	BAX	16202147, 16274043, 18074738, 19483105, 19664933
596	BCL2	18751407, 19483105, 19664933, 19751911, 19920820
6273	S100A2	17200013, 17940995
6382	SDC1	15459490, 15886501, 17455248, 18751407, 19276352
6513	SLC2A1	16096651, 19085834, 19092346, 19690016, 19819644
664	BNIP3	15856026
6678	SPARC	16041213, 17080236, 17235047
7040	TGFB1	16094523, 16101174, 16838117, 17592292, 17873896
7076	TIMP1	11773977, 12115876, 15534120, 16428484
7077	TIMP2	11181677, 11773977, 18630474
7157	TP53	19214369, 19276352, 19578815, 19664933, 19819644
7345	UCHL1	11156232, 11801558
7415	VCP	14761919, 15452376
7422	VEGFA	18612155, 18762366, 18974391, 19010843, 19389518
8434	RECK	12738734
857	CAV1	12402154, 17471232, 18802406, 19509264, 19665245
8870	IER3	18026799
898	CCNE1	10349991, 15166955, 17035403
9166	EBAG9	11992411, 12855262, 17932753
9423	NTN1	17549567
