# Supplementary material for: Google Goes Cancer: Improving Outcome Prediction for Cancer Patients by Network-Based Ranking of Marker Genes
Source: PLoS Comput Biol. 2012 May 17;8(5):e1002511. doi: 10.1371/journal.pcbi.1002511 (PMC3355064; doi:10.1371/journal.pcbi.1002511)
Supplement: Table S3 — KEGG pathways most affected by signature genes and their interaction partners. (PDF) [file pcbi.1002511.s009.pdf]

Table S3. KEGG pathways most affected by signature genes and their interaction partners.

Pathway	Number of genes	Category
Cytokine-cytokine receptor interaction	34	Signaling
MAPK signaling pathway	21	Signaling
Jak-STAT signaling pathway	21	Signaling
Focal adhesion	20	Basic cellular function
Small cell lung cancer	16	Cancer
Neuroactive ligand-receptor interaction	15	
Complement and coagulation cascades	14	
Calcium signaling pathway	13	Signaling
Hematopoietic cell lineage	13	
Toll-like receptor signaling pathway	12	Signaling
Regulation of actin cytoskeleton	12	Basic cellular function
Colorectal cancer	12	Cancer
Prostate cancer	12	Cancer
Drug metabolism - cytochrome P450	11	
p53 signaling pathway	11	Signaling
Apoptosis	11	Basic cellular function
Natural killer cell mediated cytotoxicity	11	
Pancreatic cancer	11	Cancer
Chronic myeloid leukemia	11	Cancer
Cell Communication	10	Basic cellular function
Cell cycle	10	Basic cellular function
Wnt signaling pathway	10	Signaling
